# Advancing predictive modeling in archaeology: An evaluation of regression and machine learning methods on the Grand Staircase-Escalante National Monument

**DOI:** 10.1371/journal.pone.0239424

**Published:** 2020-10-01

**Authors:** Peter M. Yaworsky, Kenneth B. Vernon, Jerry D. Spangler, Simon C. Brewer, Brian F. Codding

**Affiliations:** 1 Department of Anthropology, University of Utah, Salt Lake City, Utah, United States of America; 2 Archaeological Center, University of Utah, Salt Lake City, Utah, United States of America; 3 Global Change and Sustainability Center, Salt Lake City, Utah, United States of America; 4 Colorado Plateau Archaeological Alliance, Ogden, Utah, United States of America; 5 Department of Geography, University of Utah, Salt Lake City, Utah, United States of America; Universitat de Barcelona, SPAIN

## Abstract

Predictive models are central to both archaeological research and cultural resource management. Yet, archaeological applications of predictive models are often insufficient due to small training data sets, inadequate statistical techniques, and a lack of theoretical insight to explain the responses of past land use to predictor variables. Here we address these critiques and evaluate the predictive power of four statistical approaches widely used in ecological modeling—generalized linear models, generalized additive models, maximum entropy, and random forests—to predict the locations of Formative Period (2100–650 BP) archaeological sites in the Grand Staircase-Escalante National Monument. We assess each modeling approach using a threshold-independent measure, the area under the curve (AUC), and threshold-dependent measures, like the true skill statistic. We find that the majority of the modeling approaches struggle with archaeological datasets due to the frequent lack of true-absence locations, which violates model assumptions of generalized linear models, generalized additive models, and random forests, as well as measures of their predictive power (AUC). Maximum entropy is the only method tested here which is capable of utilizing pseudo-absence points (inferred absence data based on known presence data) and controlling for a non-representative sampling of the landscape, thus making maximum entropy the best modeling approach for common archaeological data when the goal is prediction. Regression-based approaches may be more applicable when prediction is not the goal, given their grounding in well-established statistical theory. Random forests, while the most powerful, is not applicable to archaeological data except in the rare case where true-absence data exist. Our results have significant implications for the application of predictive models by archaeologists for research and conservation purposes and highlight the importance of understanding model assumptions.

## 1.0 Introduction

Predicting how and explaining why past people used their landscape is critical for informing contemporary land management decisions [1] and answering key anthropological research questions [2–5]. To this end, archaeological researchers often create predictive models [6,7]. Archaeological predictive modeling is “the practice of building models that in some way, indicate the likelihood of archaeological sites, cultural resources, or past landscape use across a region” [8]. While this effort goes back at least as early as the 1950s [8–10], the advent of personal computers, geographic information systems (GIS), high-resolution environmental data, and robust statistical techniques has dramatically increased the implementation and creation of predictive models [8]. However, archaeological applications of predictive models still suffer from several underlying problems. Some problems are theoretical (e.g., limited *a priori* consideration of how land use decisions vary over time, a-theoretical selection of environmental variables), some are empirical (e.g., collinearity of predictor data, limited spatial resolution of environmental data, failure to account for differences in significant temporal and functional subsets of sites), and others still are analytical (e.g., inappropriate or inadequate statistical models, little to no consideration of model validation) [11–13]. Here we aim to address these theoretical, empirical, and analytical shortcomings by building on recent theoretical insights from behavioral ecology [14,15] and methodological innovations from ecology [16,17].

**Fig 1:**
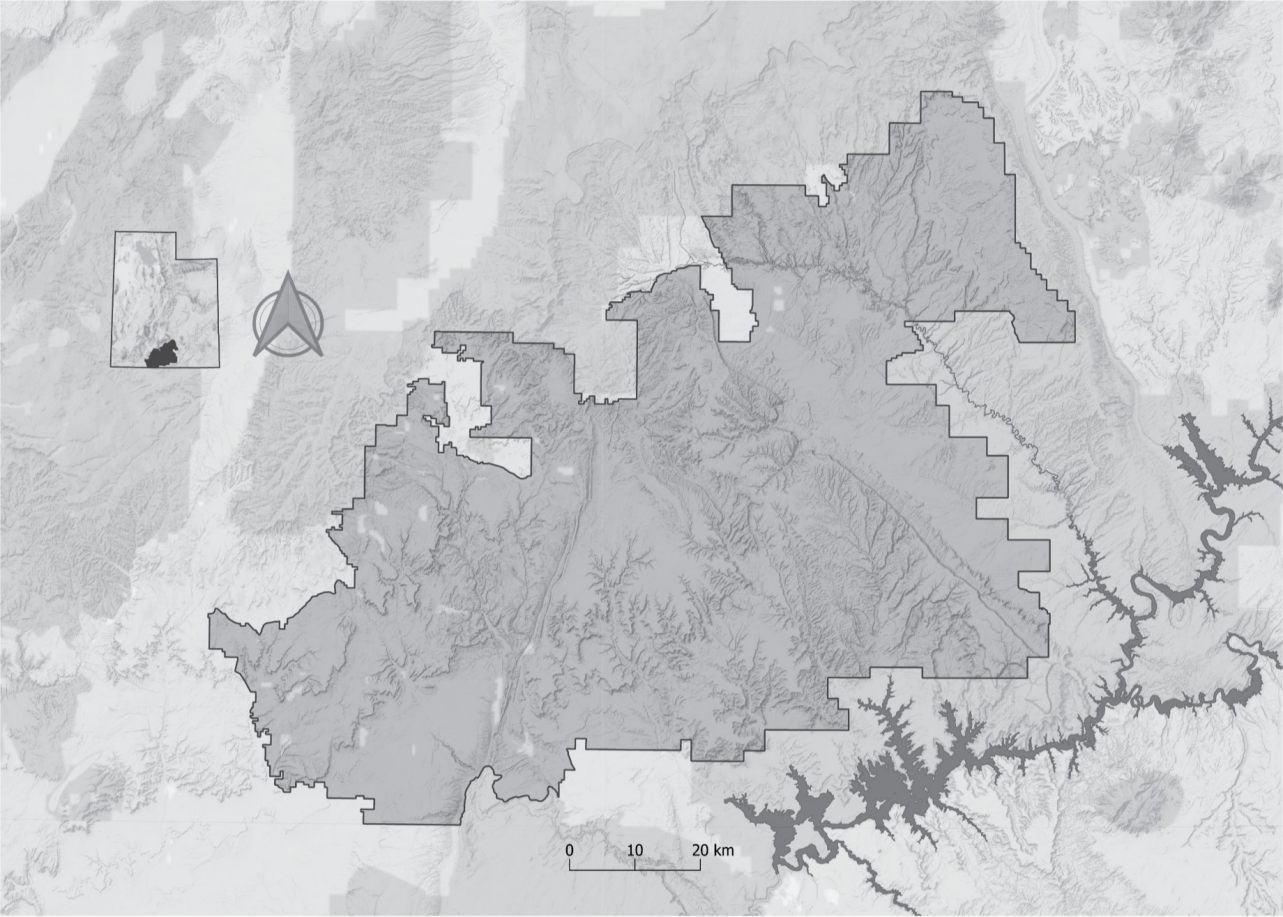
Location map of the Grand Staircase-Escalante National Monument in south central Utah, USA.

Our study relies on archaeological site data from the Grand Staircase-Escalante National Monument (GSENM) in southern Utah, USA (Fig 1). We use the original 1995 GSENM boundaries, which include an area of 1,880,461 acres (7,610 km^2^). In 2017, Executive Proclamation 9682 reduced the size of the GSENM from 1.88 million acres to 1 million acres [18]. Based on records from the Utah State Historic Preservation Office (UT-SHPO), approximately 10% of the original GSENM has been systematically inventoried for cultural resources [19,20]. This leaves approximately 1.69 million acres of un-inventoried area and highlights the need for a predictive model to make informed conservation decisions and sound environmental impact assessments of Executive Proclamation 9682 [20,21].

The study builds a foundation for local and regional research into changing land use patterns. On the local level, our research creates a structured data set of known archaeological sites and models the distribution and land use patterns of farmers in an arid landscape. Using the modeling framework here, we can address questions about the local transition to agriculture and agriculture’s effects on prehistoric population distributions on the GSENM [22]. On the regional scale, we can begin to address how arid farmers of the southwest responded to volatile climatic conditions and the subsistence-settlement dynamics that ultimately led to decreased population and a return to foraging subsistence patterns [23].

First, we limit our archaeological response data to sites from the same time period, when subsistence practices and population densities should lead to similar land use decisions (discussed in Section 2.2). Archaeological predictive models often incorporate all known site locations, regardless of period, which can lead to spurious results as past land use decisions vary through time [24]. Because it is the non-random patterning of human behavior that allows us to create predictive models in the first place [3,6,8], modeling should leverage the patterning in these past decisions to the highest degree possible and thus differentiate sites of different periods to isolate similar behavioral adaptations that should lead to similar land use decisions.

Second, we use theoretically-informed expectations to select environmental predictor data that should structure past land use [4,7]. Insights from behavioral ecology provide clear expectations about what factors should structure past land use [14,15,25–27], thereby linking underlying explanations of human behavior to the predicted outcomes (e.g., settlement decisions should maximize habitat suitability, minimize travel distance to resource patches). We then evaluate and reduce collinearity in that data to avoid redundancy and increase the predictive power of the subsequent statistical models [28]. Because these predictors may correlate with one another, we use a principal component analysis as a dimensional reduction technique to identify and reduce collinearity.

Finally, as the core of this paper, we run and cross-validate four empirical models and assess their performance using a threshold-independent measure, AUC, along with a series of threshold-dependent measures. Two of these models are standard regression approaches: generalized linear models (GLM) and generalized additive models (GAM). The other two are machine learning-based: maximum entropy (MaxEnt) and random forests (RF). While regression-based models are the statistical work-horse in archaeology [29–31], machine learning models are rarely used [32,33] but are standard for spatial prediction problems in ecology [17,34–36].

## 2.0 Materials and methods

**Fig 2:**
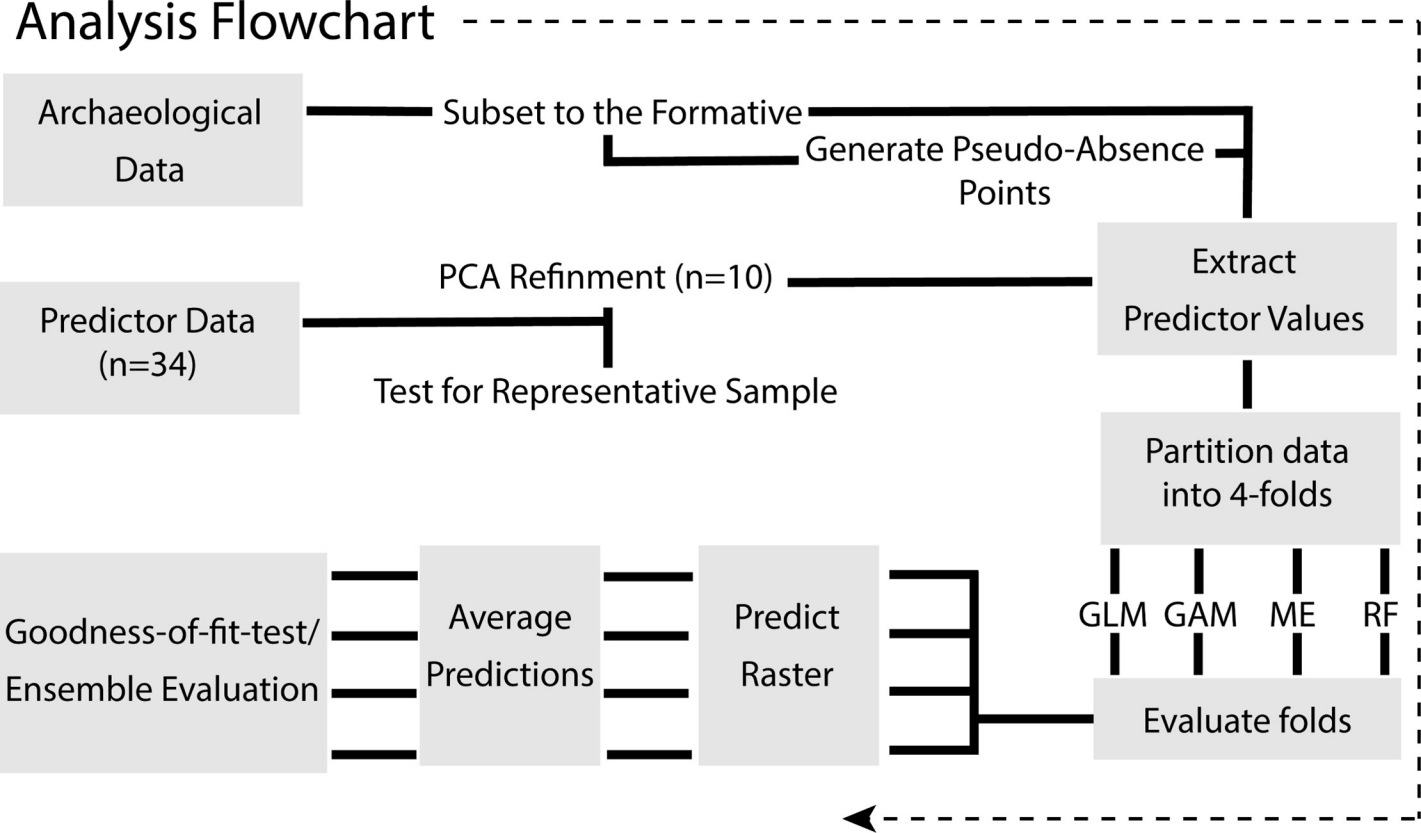
Flowchart of analysis.

Our approach has three core components. First (S 2.1), we compile response data for all known Formative Period (2100–650 BP) sites in the GSENM and generate absence points at locations with no known archaeological sites. Second (S 2.2), we draw on theory from behavioral ecology to identify key environmental predictors of past land use, and then evaluate and reduce collinearity in these predictors. Finally (S 2.3), we evaluate the efficacy of four predictive models: GLM, GAM, MaxEnt, and RF (Fig 2). The R Environment is used to run all analyses [37] and to increase the replicability and reproducibility of these analyses [38], all of the code is available in an annotated Markdown document (S1 File), along with the data (S2 File. Advancing Predictive Modeling in Archaeology–Supplementary Data) on the Digital Archaeological Record (tDAR) repository [39]. All data were collected under Utah State Public Lands Policy Coordinating Principal Investigator Permit number 328. All necessary permits were obtained for the described study, which complied with all relevant regulations.

### 2.1 Archaeological background

The Formative Period (2100–650 BP) in the GSENM is typically described within the context of two similar maize farming lifeways (see [19] for a comparison of regional organizational schemes). (1) The Virgin Branch of the Grand Staircase region shares Ancestral Puebloan material culture and architectural traits with contemporaneous groups to the south in the Kayenta area in Arizona, and to the west in the Virgin River Basin of southern Utah and southern Nevada. (2) The Fremont complex of the Escalante River drainage shares characteristics similar to those found among Utah Fremont groups farther to the north and northwest.

Maize farming was firmly established in the Grand Staircase region by about 2100 BP with the appearance of highly sedentary, agriculturally dependent communities situated alongside permanent water sources [40–42]. These residential clusters are characterized by pithouse architecture, large-capacity food storage for cultigens, and elaborate burial practices, traits that are collectively referred to in this region as Basketmaker II, a label that recognizes similarities to contemporaneous adaptations elsewhere in the greater Southwest (cf. [43]).

Farming did not take root in the Escalante River drainage until some four centuries later, at about 1750 BP [44,45]. Agriculture here simultaneously emerged alongside elaborate subterranean and surface storage strategies with implications for lower group mobility [46]. But a co-occurring pithouse tradition and residential aggregation have not yet been identified; the earliest formal pithouses in this region date to about 1200 BP, and they share remarkable similarities to Ancestral Puebloan pithouses to the west [47]. The earliest Fremont farmers instead appear to have been seasonally mobile, living in temporary brush structures and alcoves along the Escalante River and its tributaries in the summer as they tended to their crops [44], and then relocating to upland winter residences with access to wild plant resources and abundant fuelwood [48,49], and perhaps to exploit mule deer winter ranges [45].

Evidence of socioeconomic interaction between the earliest Virgin Branch communities to the west and Fremont groups to the east appears to have been minimal in early agricultural times, suggesting separate cultural identities maintained through hard boundaries that became increasingly permeable in later Formative times. These boundaries had all but disappeared by about 900 BP [50,51].

There is no consensus on the cause(s) that led to the demise of Formative lifeways by 650 BP. Climatic deterioration characterized by extended droughts between 850 and 670 BP might have been factors [52], but these farmers had survived similar climatic fluctuations in the past. If increased reliance on foraged foods had been a traditional strategy that allowed farmers to survive earlier droughts, then they might have been out-competed for those contingency resources by recently arrived Ancestral Paiute foragers [19]. But there is, as yet, minimal evidence that Formative farmers coexisted with Paiute foragers anywhere in the GSENM ([19]; see also [53] for a regional perspective).

#### 2.1.1 Response variables: Site data

We compile the archaeological site database from the digital site form data received from the GSENM Bureau of Land Management (BLM) in Kanab, Utah. The site database (GSENM-SiteDB-01) was originally generated for a Class I archaeological investigation by the Colorado Plateau Archaeological Alliance in collaboration with the University of Utah Archaeological Center [19]. The site data consist of 4,529 archaeological sites with 1,619 Formative Period sites. The Formative Period sites are identified by manually reviewing each archaeological site form, most of which are Intermountain Antiquity Computer System (IMACS) data collection forms. Our criteria for determining whether a site is Formative are based on previous archaeologists’ determination of time period and diagnostic cultural material and features documented in the data collection forms (see Sec 10.2 in S1 File). Note, we could further partition sites into interpreted functional categories, but we choose not to because this would introduce further uncertainty about whether our interpretations were meaningful, and the additional models would significantly increase computation time and analysis complexity.

*Spatial Data*. The predictive models used here are based on spatial point data. As the georeferencing for some archaeological sites is in a polygon format, these are reduced to points by estimating the centroid of the polygon.

*Documentation History*. The data in this study date from the 1920s to 2018 and were collected using a number of inventory methods. The majority of the data collected from digital site forms come from a standard recording method, IMACS. The GSENM BLM has made a concerted effort to revisit and re-document archaeological sites not yet recorded according to IMACS standards, so while original documentation for some sites may have occurred prior to the standardized format, most of the database is in an IMACS format. See Sec 10.3 in S1 File for more information. That said, the range of data collection methods and definitions pose a significant problem for archaeologists using large archaeological databases generated from decades of cultural resource management work. However, these issues are not unique to archaeology. Ecologists struggle with similar problems related to data quality and collection methods [54,55]. These most often manifest as imperfect detection, or assumed absence when, in reality, it (a species or an archaeological site) is present, and sampling bias [1,6,54–57]. The consensus in archaeology is that we attempt to make use of these large datasets by using methods that address potential biases and acknowledging the limitations of the data [1,6,23,56,58,59].

#### 2.1.2 Pseudo-absence points

Predictive models require comparisons between attributes of known site (or species) locations with attributes of absence locations. For absence locations, we generate random pseudo-absence points (or background points) in raster cells that do not have known occurrences of archaeological sites [60,61]. While we could generate true-absence points within negative inventory, the quality of negative survey data is suspect as a result of changing archaeological site documentation standards. We generate 20,000 points within the entire rectangular extent of the GSENM, and then remove points outside of the GSENM boundary polygon resulting in a total of 10,195 absence points (see Sec 3.2 in S1 File). Our target number is at least 10,000 points within the GSENM boundary [60].

### 2.2. Environmental predictor variables

Predictor variables are the explanatory or independent variables used in predictive models. Both predictive models in archaeology [30,32,56] and species distribution models in ecology [17,34–36] rely on environmental data as the predictor of site or species occurrence. The predictor variables need to reflect the socioenvironmental factors that influence land use decisions. Because changes in subsistence likely produce differences in land use patterns, we select data that pertain to the Formative Period representing agricultural-based subsistence strategies. Predictor variables align with four categories: resource distribution, climate, environmental (agricultural) productivity, and landscape attributes (see Sec 2.0 in S1 File). Each of these variable categories captures the costs and benefits individuals would have experienced when selecting where to settle. We evaluate the relative cost and benefits of each using logic from central place foraging [62], prey choice [63], and ideal distribution [26] models. All predictor variable data, including descriptions, resolution, units, and citations are found in Sec 10.1 in S1 File.

**Resource distribution.** All else held equal; we expect people to distribute themselves close to profitable resource patches to reduce travel time between resource acquisition locations and central places [62,64,65]. Predictor data for resource distribution in the GSENM primarily focuses on the distance to water, both as a necessary resource and as a proxy for other profitable resources in the arid environment that characterizes the Colorado Plateau. The resource distribution predictor variables are cost-distance maps (rasters) measured in time, which allows us to account for ways topography impedes or facilitates travel [66,67]. To create the cost-distance maps, we use Tobler’s hiking function which accounts for differential walking speeds as an effect of slope [68]. The default walking speed in our cost-distance rasters is 4 kilometers per hour. We create cost-distance maps at extents larger than the GSENM boundary to avoid an edge-effect [67].

**Environmental productivity.** The overall productivity of an environment determines the abundance of profitable resources and resource patches. Assuming that individuals seek to maximize their rate of energetic return (see [63,69]), they should prefer to occupy locations of higher environmental productivity [26]. While most often applied to hunting and gathering populations [70], this logic also holds for agriculturalists [71]. Given that the broad diets of agriculturalists [72] consisted of less profitable resources requiring significant investment in handling rather than search, they should be less mobile and, therefore, more sensitive to variation in environmental productivity [73]. As such, environmental productivity will be relevant for all time periods, but particularly important for agriculturalists.

**Climate.** Agricultural productivity is also highly dependent upon local climate [74]. As such, variation in climatic variables likely influenced the decisions of prehistoric agriculturalists on the GSENM. Our climate variables are derived from contemporary monthly and annual 30-year averages at a resolution of 800 meters [75]. Contemporary monthly and annual climate data include: temperature, precipitation, dew point, and vapor pressure deficit [75]. Using the contemporary monthly data, we calculate seasonal averages for the four seasons (see Sec 2.1.3 in S1 File). While we do not account for past climate change, we assume that any past climate variations were homogeneous across the GSENM. That is if climate became hotter and dryer by an order of magnitude in one area, it also became hotter and dryer by a similar magnitude in other areas, with the relative difference remaining constant [71].

**Landscape attributes.** Landscape attributes impose physiological constraints on land use. These data are commonly documented at archaeological sites and are recognized as potential contributors to land use patterns [30,76]. Landscape attributes include such things as elevation, slope, and aspect. Aspect, a common proxy of solar irradiance, is often measured in degrees, which can produce issues in regression-based models that interpret the degrees as linear data. To overcome this issue, we decompose aspect into two continuous variables, east aspect and north aspect (see Supplemental Section 2.2).

In total we select 55 predictor variables. We crop the predictor data to the appropriate extent and resample to match the resolution of the highest resolution dataset (DEM) at a cell size of 5 meters^2^ (see Sec 2.1.2 in S1 File). See Sec 10.1 in S1 File for a complete description of predictor variables.

#### 2.2.1 Collinearity: Predictor variable refinement using PCA

Collinearity among predictor variables is often overlooked in predictive modeling. Using predictor variables that correlate strongly can result in an overestimate of the predictive power of a model, as well as reducing the interpretability of the model parameters [28]. There are several ways to address the issue [28]. Here we use a principal component analysis (PCA) to identify predictor variables that correlate the least. We do this by looking at the principal components and eigenvalues produced by PCAs to identify what each principal component is describing in our data [28]. We select the first 10 components (see Supplemental Section 2.3), which account for 95% of the total variance. For each of these, we select a single predictor variable that has a high eigenvalue indicating a close match to what the principal component is describing. This allows us to select uncorrelated predictors while also retaining interpretability by using the original units of measure rather than the resulting principal components. Next, using a random sample of 20,000 points, we examine the correlation between the refined predictor variables to determine whether the variables selected show signs of collinearity.

#### 2.2.2. Representative sample test

To test whether the sampled area (areas where inventories have occurred) is representative of the range of possible habitats, we use a permutation test that calculates a distribution of mean-differences on a series of sample points from the predictors within and outside (extent) of the sampled area. The mean of the mean-differences distributions will have a mean close to 0 if the sampled area is representative of the range of possible habitats. If our sample falls within 95% of the extent distribution, we conclude that it is representative (see Sec 2.5 in S1 File).

### 2.3 Statistical models: Regression and machine learning

We use two types of statistical approaches, standard regression techniques, and newer machine learning approaches. Generally, the benefits of standard regression methods are that they are well established and based on robust statistical theory, which provides tools for inference [77]. The benefits of machine learning approaches are found in their flexibility, predictive power, and methods of regularization to avoid overfitting. As such, machine learning methods are powerful tools for prediction but lack the general comprehension and theoretical grounding of standard regression techniques [77].

#### 2.3.1 Regression approaches

The standard regression models used here are generalized linear models (GLM) and generalized additive models (GAM). Both perform logistic regression and fit models using maximum likelihood estimation [78,79]. The primary difference between the two is that a GAM is able to model non-linear responses of predictor variables though use of additive functions (e.g. smoothing splines) [78,79].

***GLM*.** The most prevalent predictive modeling approach in archaeology is binomial logistic regression. These models can use a combination of categorical and continuous predictor variables to model a binary or proportional outcome, for example, the presence or absence of an archeological site. Binomial models can be fit in a generalized linear model (GLM) framework [78], using a logit transformation of the response variable and a Binomial distribution of the model errors.

The logit-transform results in a model of the log-odds, rather than the raw presence/absences, and the coefficients (*β*) need to be back-transformed using an exponential function to interpret them as changes in the odds of the presence of a site. Models are fit by maximizing the likelihood that the model parameters match the observed set of presences and absences [78]. With archeological site data, these models, therefore, consider the absences to be true, observed absences. With this assumption in mind, the models can be used, once fit, to estimate the probability of site occurrence at any new location where the predictor variables are known.

***GAM*.** A generalized additive model (GAM) extends the GLM approach to include non-linear relationships between a response variable and covariates [79]. While these may be approximated using polynomial transformations of the response variables in a GLM, GAMs use spline functions (e.g. smoothing or B-splines), which do not need the form of the relationship to be pre-specified in the model. For any response and predictor variable, a spline is a series of polynomial functions that added together form a smoothly varying function that follows the non-linear relationship. The smoothness of the spline is an important factor as more complex splines will over-fit to the data, including to the noise the relationship between the two variables. This is limited in GAMs, by using cross-validation to choose spline complexity. The final model replaces the beta coefficients (linear predictors) found in GLM with a response function that describes the varying link between the response and predictors [79].

#### 2.3.2 Machine learning approaches

Machine learning approaches rely on iterations, permutation tests, and regularization [80], and as a result, are generally more difficult to understand. Machine learning methods are generally fit to data in an iterative fashion; a model is built, often based on simple or random parameter choices, and these are gradually ‘tuned’ to the data by minimizing an error or loss function. The nature of this iterative approach often results in overfitting the model to the data, with the result that generalization or prediction becomes increasingly difficult. Overfitting can be prevented by regularization, in which individual variables are down-weighted or removed [81], and/or by ensemble methods, which use weak models constructed using different permutations of the data [82]. The resulting models avoid the issue of overfitting while demonstrating a predictive power that is frequently superior to classical regression [80]. The machine learning approaches used here are Maximum Entropy (MaxEnt) and Random Forests (RF).

***MaxEnt*.** MaxEnt begins with the assumption of a uniform distribution in which all locations are equally likely to have a presence point (i.e., a distribution of maximum entropy) [83]. For each environmental variable, this forms a probability density function across its range, where the height or density of the function is proportional to the frequency with which that value of the variable exists on the landscape. Predictor variables are then used to constrain this distribution by increasing the density at values of the variable at presence locations. The resulting probability is then a trade-off between these: if 10% of the presence locations occur at an environmental value that occupies 10% of the study area, this value will have the same overall probability as a value with that has only 1% of the sites and occupies 1% of the area. If this second value has 2% of the sites, then the overall probability will be double that of the first value, despite having many fewer presence sites. The constraints imposed by the predictors and the principle of maximum entropy then work against each other. The constraints work to create a non-uniform distribution, while entropy works towards a uniform distribution [17]. By retaining the most uniform distribution possible with the constraints imposed by the predictor variable values, MaxEnt creates the least biased and most conservative estimate of presence or retains the distribution with the greatest entropy [84].

A key component of MaxEnt is how it incorporates the constraints from the predictor variables. The distributions described above are modeled using a set of “features” [85]. Feature sets are created from the set of predictor variables, and may include linear and non-linear transformations as well as interactions between variables. The model is initialized with a uniform distribution for each feature, i.e., the probability at each value is just based on the frequency of that value in the landscape. A set of coefficients are then randomly generated, and the fit to the presence sites is estimated using a log-likelihood function. The coefficients are then adjusted using a random walk in parameter space, and the log-likelihood re-estimated. If the fit improves, the new coefficient values are retained. If not, they are rejected. Regularization is used to prevent overfitting by limiting the increase in the log-likelihood. This has the effect of relaxing the constraints, and ultimately rejecting certain features (and even variables) if they do not improve the model. The raw output for MaxEnt is raw probabilities, which sum to one across the region and represent the relative rate of occurrence on the landscape. This is converted to log-odds and then probabilities for interpretability [86]. The underlying model fitting is rooted in generalized linear models, but with the addition of features which allow for more complex variable responses and interactions [86–88].

***Random Forests*.** RF is based on classification and regression tree (CART) analysis, a non-linear, non-parametric approach to modeling data. In the CART method, predictor variables are used to identify thresholds that partition the dataset into smaller subsets, in which the value of the response variable should be as constant or homogenous as possible. The method is recursive, so each subset formed can then be partitioned into two further subsets, creating a decision tree. Predictions can then be made for a new observation by finding which of these subsets it belongs to. The repeated recursion leads easily to overfitting, and one issue with the use of CART is developing a stopping rule: a decision of when to stop splitting the dataset. Because of this, CART decision trees are said to have high variance, i.e., the resulting model is unstable and highly dependent on the dataset used.

RF [89] overcome this by developing many individual trees, each based on a bootstrapped resample of the original data. Predictions for new cases are then made as the average of all trees for continuous variables, and as the most common class for categorical variables. While each individual tree may be highly biased and have high variance, the ensemble has low variances and is much less susceptible to changes in the dataset [82]. The number of trees used in RF is determined by the user, but generally, several hundred are created. As each tree is effectively independent from all others, there is no risk of overfitting by creating too many trees. To prevent correlation between trees, i.e., all trees being dominated by a single covariate, only a subset of variables is used to make any single split in a tree [89]. The number of predictor variables used in each tree is dependent on whether it is classification (*sqrt(m)*) or regression (*m/3*) where *m* is the number of predictors. While binary variables can be treated as a classification problem, Hijmans and Elith [90] recommend treating the prediction of species distributions as a regression problem, and we follow this here, resulting in each tree determining a probability of occurrence. The final predicted probability is then made by averaging across all decision trees in the forest.

### 2.4 Model evaluation/validation

To adequately assess the different modeling approaches, we use four-fold cross-validation. We choose four-folds to balance computational time and retain adequate training and test sample sizes [91]. Each fold has approximately 2,954 observations (433 sites and 2521 pseudo-absence points). The same folds are used in each modeling approach to remain comparable.

**Fig 3:**
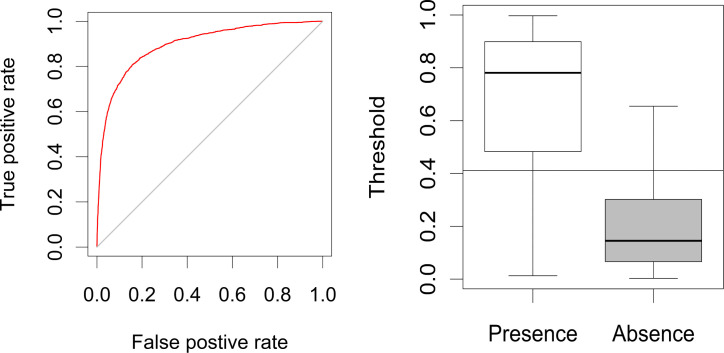
**Left:** Receiver operating characteristic (ROC) for MaxEnt. The ROC presents the trade-offs between the FPR and TPR at different thresholds. The area under the ROC curve (AUC) is a measure of the model’s ability to successfully discriminate between presence and absence points for a range of probability thresholds. The diagonal line represents an AUC value of 0.5, equivalent to randomly guessing whether an observation is a presence or an absence. An AUC of 1 would indicate that at some threshold, the model can discriminate between the two classes perfectly. **Right:** The predicted probabilities by the ensemble MaxEnt model for presence and absence points. The horizontal line lies at the optimal threshold (0.411). The figure demonstrates how many presence and absence points are correctly predicted as presence points if we were to convert the probabilities to binary certainties using the optimal threshold.

We evaluate model results using a threshold-independent measure, the area under the Receiver Operating Characteristic curve (AUC) [17,91]. The output of a predictive model is a continuous probability corresponding to the likelihood of a positive occurrence [17], so to gauge a model’s performance, we need to know something about how well it does at correctly identifying presence from absence. The number of presences and absences correctly identified is dependent on what the threshold is for classification. We can think of these as rates of correct classification [true positive rate (TPR) and true negative rate (TNR)] and incorrect classification [false positive rate (FPR) and false negative rate (FNR)]. Because the distribution of probabilities over presence and absence locations are model specific, the degree to which TPR and TNR fluctuate will also be model specific [91]. To make modeling approaches comparable, it is necessary to use an evaluation measure that is independent of the threshold [17]. AUC is an effective threshold-independent measure that allows us to compare modeling approaches [17]. AUC evaluates model performance using the TPR and TNR at every threshold [17,92] (Fig 3).

Measures of performance dependent on threshold values are known as threshold-dependent evaluation methods [17,92]. Threshold-dependent evaluation methods verify that a model performs better than random by using a set threshold value to classify values from the continuous probability distribution as predicted presence or absence [91]. Because commonly used binary maps require a threshold selection, here we calculate an optimal threshold. The optimal threshold is the threshold where the TPR and TNR are maximized [91]. Next, we derive a series of threshold-dependent measures, including the true-skill statistic (TSS), kappa, and several commonly used archaeological threshold-dependent measures (See Sec. 8.5 in S1 File) [6]. Threshold-dependent statistics are specific to selected model thresholds and are not comparable between models unless a standard method of threshold selection is used [91,92].

Analysis of variance (ANOVA) is used to determine whether differences exist between the performance of the four modeling approaches and the post-hoc Tukey HSD test is used to identify pairwise differences in AUC between modeling approaches.

### 2.5 Ensemble predictive maps

For each modeling approach, we generate four predictive maps (one per fold of data). The corresponding raster cells in the four predictive maps are then averaged to create a final predictive map for each modeling approach. To evaluate this final predictive map, we use all observations (presence and absence) to evaluate the goodness-of-fit. Because we use all of the data to assess the fit of the predictive map, in this application, the AUC is a measure of the goodness-of-fit.

To create binary maps, we use the optimal threshold determined by the probability at which the sum of the true-positive rate (TPR) and true-negative rate (TNR) is maximized, and the predictive skill of the binary maps was assessed using the TSS and other standard threshold-dependent measures used in archaeology [6,60,92].

## 3.0 Results

The full dataset is comprised of 11,814 observations (1,619 presence points and 10,195 absence points), with 55 predictor variables.

### 3.1 Predictor variable refinement using PCA

Using the PCA, we identify ten predictor variables with little collinearity (less than 0.7) [28]. At principal component 10, 95% of the variance within the data is explained. Based on the PCA, we select ten predictor variables that each correlate highly with one of the components (Table 1).

**Table 1:** Attributes of the ten predictor variables that have limited collinearity as identified by the PCA. See Sec. 10.1 in S1 File for more information on these data.

Predictor Variable	Category	Strongest Correlation
East-West Aspect	Landscape Attribute	-0.027
North-South Aspect	Landscape Attribute	-0.038
Slope	Landscape Attribute	0.213
Watershed Size	Landscape Attribute	0.252
Mean Temperature	Climate	0.371
Net Primary Productivity	Environmental Productivity	-0.112
Growing Degree-days	Environmental Productivity	0.371
Cost-distance to Springs	Resource Distribution	0.422
Cost-distance to Streams	Resource Distribution	0.178
Cost-distance to Wetlands	Resource Distribution	0.422

The PCA does well at selecting predictor variables with low correlation among other variables (Pearson correlation coefficient, min = 0.0, max = 0.422, mean = 0.063, sd = 0.092) (see Sec 2.4 in S1 File). The strongest correlation (p = 0.422) is between cost-distance to wetlands and cost-distance to springs.

#### 3.1.1 Representative sample test results

The permutation tests of mean-differences reveal that our sampled area is not representative of the possible raster values in the study area for five of our predictor variables (see Sec 2.5 in S1 File). At a threshold of 0.05, we have a representative sample for: east aspect (p = 0.649), cost-distance to springs (p = 0.296), north aspect (p = 0.279), mean annual temperature (p = 0.083), and GDD (p = 0.637) and a biased sample for watershed size (p<0.001), cost-distance to wetlands (p = 0.002), cost-distance to streams (p = 0.006), slope (p = 0.004), and net primary productivity (p = 0.007). The results show that there is greater variation in the study area for these five variables than is represented by the presence points.

### 3.2 Model results

**Fig 4:**
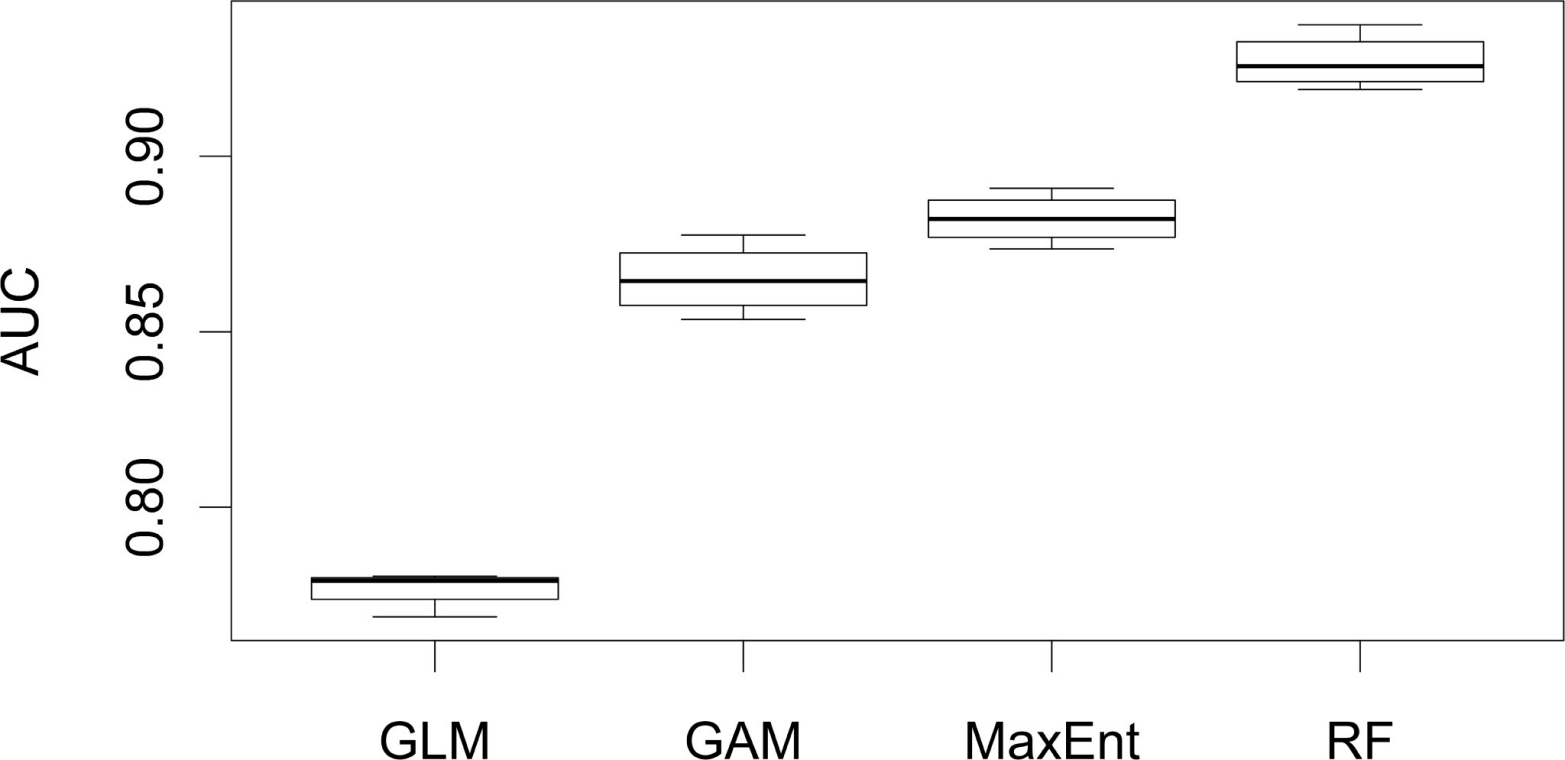
Results of four-fold cross-validation. In column one, we represent the four modeling approaches. Column two shows which partition of the four-fold cross validation is used. Columns three and four show the AUC and optimal threshold of each fold and column 5 shows which variable contributes most to model predictions.

Individual model results are found in Table 2. More details on individual model performance are found in Sec 6 in S1 File. The RF AUC values are consistently higher than all other modeling approaches. The MaxEnt and GAM AUC statistics are similar, with slightly higher AUC scores for MaxEnt. The GLM approach produces consistently lower AUC statistics than the other modeling approaches (Fig 4).

**Table 2:** The four-fold cross-validation results identify GLM as the least powerful and RF as the most powerful. All models produce an AUC, an optimal threshold (Opt Thresh.), and are capable of identifying the variable that contributes most to prediction (CV), but the machine learning methods often lack standard regression measures.

Model	K-fold	AUC	Opt Thresh.	CV
GLM	1	0.78	0.149	GDD
GLM	2	0.779	0.168	GDD
GLM	3	0.778	0.134	GDD
GLM	4	0.768	0.144	GDD
GAM	1	0.878	0.175	Mean Temp
GAM	2	0.867	0.146	Mean Temp
GAM	3	0.861	0.099	Mean Temp
GAM	4	0.853	0.177	Mean Temp
MaxEnt	1	0.891	0.315	Mean Temp
MaxEnt	2	0.874	0.402	Mean Temp
MaxEnt	3	0.884	0.289	Mean Temp
MaxEnt	4	0.88	0.336	Mean Temp
RF	1	0.938	0.157	Mean Temp
RF	2	0.924	0.197	Mean Temp
RF	3	0.928	0.15	Mean Temp
RF	4	0.919	0.169	Mean Temp

### 3.3 Model comparison

Comparing differences in AUC with ANOVAs shows a difference in modeling approaches (F = 364.1; df = 5; p < 0.001). The Tukey HSD test shows a significant increase in AUC from GLM to GAM (p <0.001), GAM to MaxEnt (p = 0.028), and MaxEnt to RF (p<0.001) (Table 3).

**Table 3:** Using an ANOVA and then a post-hoc Tukey HSD test, we see significant improvements at a significance threshold of 0.05 between standard regression approaches and machine learning approaches.

	GLM	GAM	MaxEnt	RF
GLM	-	<0.001	<0.001	<0.001
GAM	-	-	0.028	<0.001
MaxEnt	-	-	-	<0.001

### 3.4 Final predictive maps

**Fig 5:**
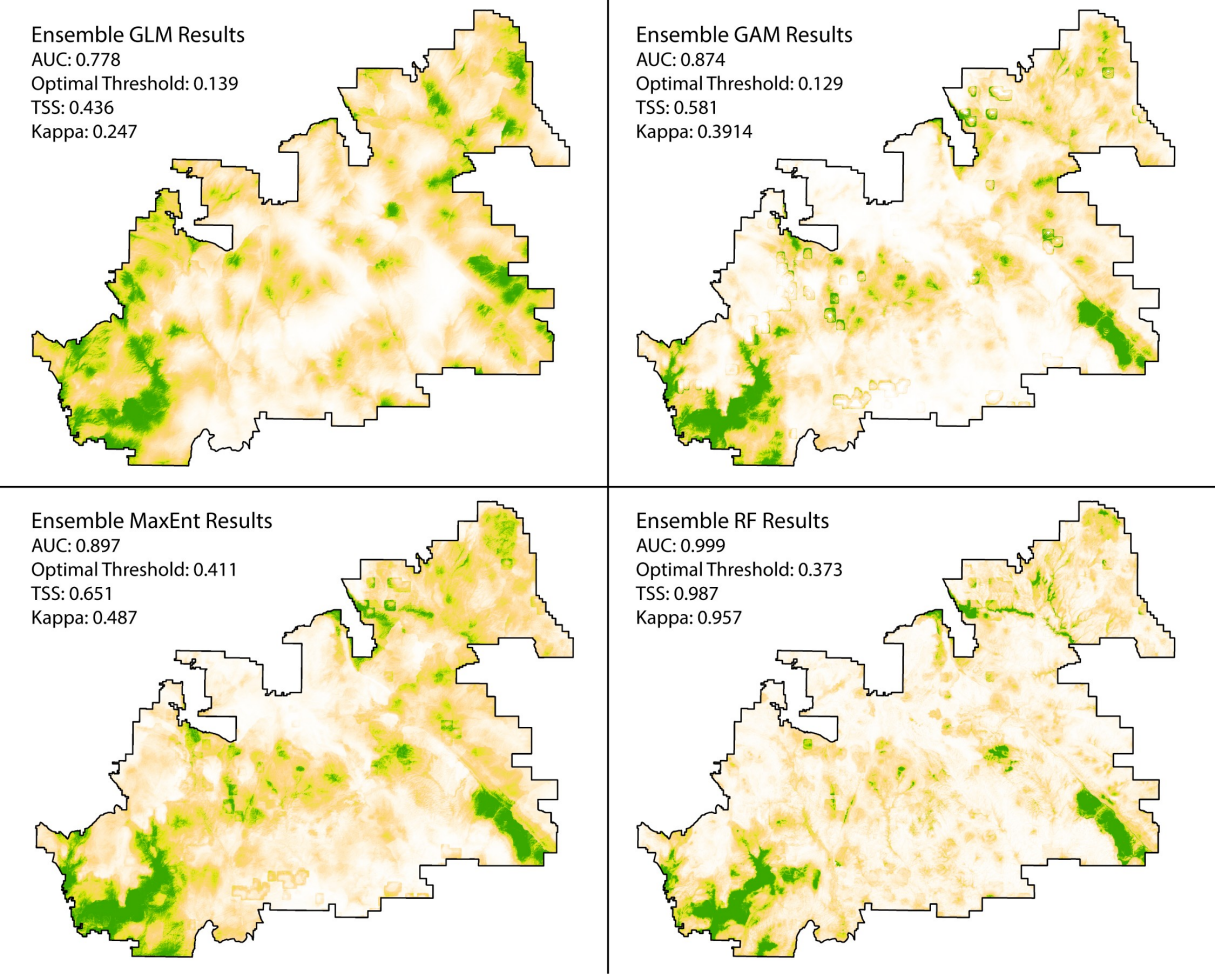
Ensemble maps using the predictions from each approach’s four-fold cross-validation. AUC and optimal threshold are determined using all presence and absence points, making the AUC a measure of goodness-of-fit. The optimal threshold is then used to determine the True-skill Statistic (TSS) and Kappa. AUC is a threshold-independent measure, while TSS and Kappa are threshold-dependent measures.

Using all of the data, we calculate AUC on each of the final predictive maps generated from averaging the results of each individual method, resulting in a predictive map for GLM, GAM, MaxEnt, and RF. Because we use all of the data to assess the fit of the predictive map, in this application, the AUC is a measure of the goodness-of-fit. The results are consistent with the model results above, with RF performing the best (Fig 5).

#### 3.4.1 Binary maps

After converting the ensemble predictive maps to binary using the optimal thresholds, we use the threshold-dependent measures, such as the TSS and Kappa statistics, to assess how each binary model performs (Fig 5; See Sec 8 in S1 File for the binary maps). The Kappa statistic is the most widely used measure for the performance of models generating presence-absence predictions, but it is influenced by prevalence. The True-skill Statistic corrects for Kappa’s dependence on prevalence [92]. Additionally, we provide threshold dependent measures commonly used by archaeologists (See Sec. 8.5 in S1 File for model evaluations) [6,33]. All threshold-dependent measures support the conclusions drawn from the threshold-independent measure, AUC.

## 4.0 Discussion

Using the 1,619 Formative Period sites (presence points), 10,195 pseudo-absence points, and our refined sample of predictor variables, we find that RF has the highest predictive skill, followed by MaxEnt, GAM, and GLM. The ensemble predictive maps reflect the results of the cross-validation, with RF achieving a near-perfect AUC.

RF has the highest AUC and is the most powerful of the predictive models, but a closer look reveals two significant violations of assumptions. The first is that GLM, GAM, and RF all assume that pseudo-absence points are true-absence points. The second is that our measure of performance, AUC (along with the threshold-dependent measures), makes the same assumption. These violated assumptions have a significant effect on determining which approach is best for predictive modeling in archaeology. In contrast, MaxEnt uses the pseudo-absence points to represent the regional environment, to which the presence points are compared. As a result, MaxEnt is more suitable for archaeological data than the other predictive modeling approaches, although the performance metrics will still be biased (see below).

### 4.1 Archaeological data and pseudo-absence points

Recognizing the strengths and weaknesses of the data is vital during model selection and evaluation. Archaeological data are often derived from resource management projects and, as such, do not represent a systematic sampling of the landscape, which leads to unquantifiable inaccuracy in negative survey results. The result is that true-absence points are often unreliable. Pseudo-absence points allow us to admit the limitations of our data by stating that presence or absence at the location is unknown [60,61]. Unfortunately, GLM, GAM, and RF assume the use of true-absence points. As such, we violate the basic assumptions of these approaches when using them.

### 4.2 AUC

The AUC score is the most widely used metric to assess and compare models of binary or categorical outcomes but has some issues in its use with models that use pseudo-absence points [93]. As AUC is calculated using all possible threshold values and the associated TPR and FNR (S 2.4), we do not have to make arbitrary choices as to the value of the threshold in discriminating presence and absences. However, AUC assumes that absences reflect true-absence points [94], and so will be biased where pseudo-absence points are used in its calculation, including all methods considered here. If this bias is constant for all methods, then the relative ranking of AUC scores still provides a basis for comparison. However, this is currently unknown and warrants further investigation, together with the creation of unbiased comparison metrics based on presence data alone [94].

### 4.3 Modeling approaches

GLM, GAM, and RF all assume true-absence points, but why then do their AUC scores differ so greatly? GLM and GAM are both standard regression techniques based on logistic regression with a binomial error term [78,79]. During model fitting, both approaches fit using maximum likelihood, which is determined on the set of presence points and assumed true-absence points. As pseudo-absence points may include unknown presence points, the model coefficients and likelihood estimates of GLM and GAM may be biased. The jump in predictive power between GLM and GAM is a result of GAM allowing for non-linear responses of predictor variables, making it more applicable to complex ecological data, but also prone to overfitting without careful selection of model parameters [79].

RF also assumes true-absence points but has a much higher AUC than GLM or GAM. The results suggest that if we had true-absence data, RF would outperform the other approaches considerably. As the main difference in approach is that RF is based on a partitioning method, it seems likely that this is accounting for threshold effects in the response of the presence data to the covariates. RF differs from GLM, GAM, and MaxEnt in that it has no underlying assumptions about distributions when model fitting [89]. However, its flexibility, simple model fitting process and assumption of true-absence points may result in over-specification when using pseudo-absence points. Unlike GLM and GAM, which also assume true-absence points, RF is not constrained by any assumption of underlying distributions. As such, it will search out model fits that maximize AUC by attempting to assign low probability values to all pseudo-absence points. While RF limits overfitting when correctly parameterized [36,89,95], the near-perfect ensemble AUC score (0.999) suggests that it may be over-specified, with both calibration and validation influenced by the assumption that the absence data are true absences. While the most powerful of the predictive modeling approaches used here, RF may not be appropriate for predictive modeling in archaeology except in the rare case where true-absence data exist with a representative sample.

MaxEnt differs from GLM, GAM, and RF in that it is capable of incorporating the pseudo-absence points without any assumption of certainty [60,61]. By retaining the most uniform distribution possible with the constraints imposed by the predictor variables, the resulting predictions are the least biased and most conservative estimate of presence [84]. Additionally, MaxEnt is capable of using the pseudo-absence points to cope with a non-representative sample of the predictor variables. It does so by using the pseudo-absence points to sample the range of possible predictor values. Response functions are formed by weighting predictor values where sites occur by how common that value is across the entire study area, irrespective of presences or absences [17]. If a predictor value is a relative rarity, but always has a presence point where observed, these values will be up-weighted, resulting in a higher probability of occurrence. If a predictor value is common, and presence points rarely occur, these values will be down-weighted so that they have a lower probability of occurrence [86].

### 4.4 Best approach?

The limits of archaeological data are twofold: non-representative sample of the full range of variation and a lack of true-absence data. The only modeling approach capable of handling these limitations, and with the fewest violated assumptions, is MaxEnt. MaxEnt can account for both of these problems through its implementation of pseudo-absence points. While still potentially problematic, archaeological best practice is to fully understand and admit the limitations of our data and be conservative with our predictions. With this in mind, when the goal is prediction with standard archaeological data, MaxEnt is currently the proper tool for archaeological predictive modeling.

### 4.5 Implications

Our findings have significant implications for archaeological resource managers and researchers. For archeological resource managers, the results provide a step-by-step method for constructing and validating robust archaeological predictive models using data gathered mainly as a part of compliance. In addition, the findings contribute to ongoing management decisions about the Grand Staircase-Escalante National Monument, particularly the effects of Executive Proclamation 9682 [18]. Whether cultural resources were considered in the decision is central to ongoing litigation [21].

For researchers, the findings highlight issues with commonly used approaches in archaeological site predictive modeling. We find that the machine learning methods outperform standard regression models, but their suitability depends on the data and the question the researcher hopes to address. The machine learning methods are better for prediction, but they lack transparency and standard statistical inference methods [77]. Using the methods showcased here, we can address local and regional research questions pertaining to changing prehistoric land use patterns, such as the transition to agriculture and agriculture’s effects on prehistoric population distributions on the GSENM and how farmers of the arid southwest responded to volatile climatic conditions and the subsistence-settlement dynamics that ultimately led to decreased population and a return to foraging subsistence patterns [22,23].

## 5.0 Conclusion

The importance of predictive models in research and resource management applications cannot be understated. When done correctly and with transparency, predictive models can help us understand past human land use patterns and develop methods of conservation and protection. Here, we provide a set of methods for better understanding potential issues researchers and resource managers will encounter when constructing a predictive model, such as identifying collinearity among predictor variables, determining how representative inventory data are of the landscape, and gauging a model’s predictive power. At the core of the paper, we compare four modeling approaches, two regression methods, and two machine learning methods. Rather than selecting the modeling approach with the greatest predictive power (RF), our results highlight the importance of understanding the limitations of archaeological data primarily generated through resource management inventory.

Regression models based on logistic regression are standard in archaeology, particularly GLMs [30,31]. While GLMs are relatively easy to understand and implement in a variety of software, our results show that they are not appropriate for predictive modeling in archaeology due to their inability to model non-linear responses. GAMs are a regression method that overcomes some of the shortfalls of GLMs. GAMs are capable of incorporating a smoothing function, which allows for non-linear responses between predictor variables [78]. GAMs perform better at predicting archaeological sites but should be used with caution because they tend to overfit. In addition, both GLM and GAM assume the use of true-absence points.

Machine learning methods like RF and MaxEnt show significant improvements in predictive power over regression-based models. RF is a machine learning method based on regression trees. It is the most powerful predictive model used in our analysis [89]. That said, it may not be the best choice in many archaeological predictive models as a result of archaeological data. RF is better suited to models with true-absence points. MaxEnt may not be as powerful as RF (although AUC assumes true-absence points as well, making the MaxEnt AUC difficult to compare) but outperforms GLM and GAM.

We find that changes in archaeological data collection standards and inventory methods only imply absence data, making it difficult to accurately predict archaeological site occurrences. Many modeling approaches, like GLM, GAM, and RF assume the use of true-absence points, but true-absence points are rare in archaeological inventory data gathered over decades for cultural resource management. Cultural resource management inventories are project-driven and generally do not focus on systematically sampling the broader landscape. As such, using a modeling approach that assumes true-absence points (like GLM, GAM, or RF) is inherently crippled by violated model assumptions. The violation of assumptions is sharply defined in our RF results, wherein RF is capable of classifying all pseudo-absence points as low probability/absence.

There are two potential solutions to the issues presented by common archaeological data. The first is to develop inventory strategies that result in true-absence data and a representative sample of the landscape or use a modeling approach that does not assume true-absence points and attempts to account for bias sampling of the landscape. Both have their issues and trade-offs. The first solution is costly, time-consuming, and potentially impossible, depending on the size of the area of interest. Although generating perfect data with a developed inventory strategy would result in a powerful and accurate predictive model, such a model is beyond the budgets and time allotments of most researchers and resource managers. The second solution utilizes existing archaeological inventory data for a region. It is cost and time effective for both researchers and resource managers while acknowledging the shortcomings of archaeological data. The trade-off is that the model will be more conservative in its estimates of likelihood and may be limited in its ability to predict in areas with greater bias in landscape sampling.

The first approach is the best, but it is beyond the reach of many researchers and resource managers. The second is possible using the MaxEnt modeling approach. Using the MaxEnt approach and other methods applied here, modelers can be explicit about the limitations of the existing data and the effects on model predictions.

MaxEnt is surprisingly adept for archaeological data. Pseudo-absence points represent archaeological data well in that archaeological inventory is often non-representative and results in confident presence data, but implied absence data. MaxEnt’s use of pseudo-absence points allows us to maintain caution with negative survey. Additionally, MaxEnt, with its use of pseudo-absence points, can potentially overcome the common issue of non-representative samples, again a problem originating from inventory data.

The methods outlined here are a step towards advancing predictive modeling in archaeology. Like applications of predictive models in ecology, archaeology is plagued by data issues. Some of these we can compensate for by using pseudo-absence points methods meant to use pseudo-absence points properly (MaxEnt), but this requires archaeologists to be conscious of the data they use in model construction. Moving forward, archaeologists need to be critical of the hidden assumptions in predictive models and move away from standard logistic regression with linear models.

## Supporting information

S1 File Supplementary material. (ZIP)

S2 File Advancing predictive modeling in archaeology–Supplementary data. (CSV)
