# Supplementary material for: Advancing predictive modeling in archaeology: An evaluation of regression and machine learning methods on the Grand Staircase-Escalante National Monument
Source: PLoS One. 2020 Oct 1;15(10):e0239424. doi: 10.1371/journal.pone.0239424 (PMC7529236; doi:10.1371/journal.pone.0239424)
Supplement: S1 File — (ZIP) [file pone.0239424.s001.zip › Supplementary_MethodsPaper_2.html]

Supplementary Material


# Supplementary Material

#### Peter M. Yaworsky

# 1 Introduction

Welcome! This document is the supplemental material accompanying the Yaworsky, Vernon, Brewer, Spangler, & Codding **Advancing Predictive Modeling in Archaeology: An evaluation of regression and machine learning methods on the Grand Staircase-Escalante National Monument**! Here, we provide a thorough walk-through in R of the methods implemented in the research. Archaeological site location data are excluded to protect the resources and some of the predictor data creation are done seperately in ArcMap.

It is important to note, that in running these analyses on your own, individual results may differ as a result of differences in random sampling and data partitioning.

The data provided are setup so that you can view R objects created within the analysis without running the R code. Example: `summary(FR.glm_1)`. First, you will need to load the Rdata file that contains all elements (see below after package installation).

To your left you will see the Table of Contents. You can navigate this document by clicking on sections of the Table of Contents. These sections correspond to sections referenced in the journal article.

The material presented in this supplmentary document will cover all analyses covered in the paper in depth, and a few other analyses not covered in the paper.

## 1.1 Required R Packages

The required R packages are below. If these are not already added-on to your R packages, you will need to install them. More information on individual packages can be found using R’s CRAN.

Last, we will load the RData file which has all of our results. While you can run almost all of the R code in the document, we suggest loading our RData file. This will save you the time of rerunning analyses, many of which are processer intensive. If you have trouble loading the RData file, you likely need to set your working directory to the file location of the RData file.

```
library(foreach)
library(rgdal)
library(raster)
library(sp)
library(maptools)
library("rJava")
library(rgeos)
library(snow)
library(mgcv)
library(parallel)
library(randomForest)
library(xgboost)
library(dismo)
library(dplyr)
library(Matrix)
#Load the data
load("./Supplementary_RDATA_NoSiteLocation.RData")
```

```
## Warning: namespace 'pROC' is not available and has been replaced
## by .GlobalEnv when processing object 'test1'
```

## 1.2 Spatial Data and R

R is amazingly versatile and can handle spatial data in much the same way that ArcGIS and GRASS can. The interface is a little more involved, but all the tools are present. Here is my favorite quick cheatsheet: (http://www.seascapemodels.org/data/ArcGIS\_to\_R\_Spatial\_CheatSheet.pdf)

As an example, here is the shapefile boundary for the GSENM:

```
#loading the shapefile
GSENM_B<-readShapePoly('./Data/GIS/Shapefiles/GSENM_B/GSENM_boundary')
```

```
#Info about the shapefile
GSENM_B
```

```
## class       : SpatialPolygonsDataFrame 
## features    : 1 
## extent      : 369866.6, 502355.2, 4095490, 4207667  (xmin, xmax, ymin, ymax)
## crs         : +init=epsg:26912 +proj=utm +zone=12 +datum=NAD83 +units=m +no_defs +ellps=GRS80 +towgs84=0,0,0 
## variables   : 10
## names       :          AREA,     PERIMETER, AOMBP_GS24, AOMBP_GS_1, NM_CASEFIL,                                     NM_NAME, NM_STATE,                MBP_DATA,         ACRES, NAME_SHORT 
## value       : 1880629.49445, 834530.313016,          2,         27,   UT000001, Grand Staircase-Escalante National Monument,       UT, GS-E NATIONAL MONUMNENT, 1880629.49445,      GSENM
```

```
#if we want to look at the feature attributes
GSENM_B@data
```

```
##      AREA PERIMETER AOMBP_GS24 AOMBP_GS_1 NM_CASEFIL
## 0 1880629  834530.3          2         27   UT000001
##                                       NM_NAME NM_STATE                MBP_DATA
## 0 Grand Staircase-Escalante National Monument       UT GS-E NATIONAL MONUMNENT
##     ACRES NAME_SHORT
## 0 1880629      GSENM
```

```
#Specifying the CRS (coordinate reference system)
proj4string(GSENM_B)<-CRS("+init=epsg:26912")
```

```
## Warning in `proj4string<-`(`*tmp*`, value = new("CRS", projargs = "+proj=utm +zone=12 +datum=NAD83 +units=m +no_defs")): A new CRS was assigned to an object with an existing CRS:
## +init=epsg:26912 +proj=utm +zone=12 +datum=NAD83 +units=m +no_defs +ellps=GRS80 +towgs84=0,0,0
## without reprojecting.
## For reprojection, use function spTransform
```

```
#plotting
plot(GSENM_B)
```

In the above, we load the boundary shapefile into R. When we call on the object, we see that it is a polygon with 1 feature, its spatial extent, its CRS (coordinate reference system), the names of the columns in the attributes tables, and some of the values. If we want a closer look at the attributes table and variables we can call on `GSENM@data`. This will print the attributes table. If we want to specify the CRS either because it doesn’t have one or we want to be sure it is in the proper CRS, we can specify the slot within the shapefile data for `proj4string()`. To plot shapefiles you can use the base `plot` function in `R`.

As an example for rasters, we will use the aspect data:

```
#loading raster
aspect<-raster("./Methods_Paper/Predictors/Final/Aspect_bin.tif")
#transforming from 1 projection to another
aspect<-projectRaster(from=aspect, crs=CRS("+init=epsg:26912"), method="ngb")
```

```
#information
aspect
```

```
## class      : RasterLayer 
## dimensions : 22436, 26498, 594509128  (nrow, ncol, ncell)
## resolution : 5, 5  (x, y)
## extent     : 369867.5, 502357.5, 4095488, 4207668  (xmin, xmax, ymin, ymax)
## crs        : +init=epsg:26912 +proj=utm +zone=12 +datum=NAD83 +units=m +no_defs +ellps=GRS80 +towgs84=0,0,0 
## source     : D:/Desktop/Grants/CPAA_GSENM/GSENM/Methods_Paper/Predictors/Final/east_west_asp.tif 
## names      : east_west_asp 
## values     : -1, 1  (min, max)
```

```
plot(aspect)
```

```
## Warning in showSRID(uprojargs, format = "PROJ", multiline = "NO"): Discarded datum North_American_Datum_1983 in CRS definition,
##  but +towgs84= values preserved
```

```
## Warning in showSRID(uprojargs, format = "PROJ", multiline = "NO"): Discarded datum Unknown based on GRS80 ellipsoid in CRS definition,
##  but +towgs84= values preserved
```

```
## Warning in showSRID(uprojargs, format = "PROJ", multiline = "NO"): Discarded datum North_American_Datum_1983 in CRS definition,
##  but +towgs84= values preserved

## Warning in showSRID(uprojargs, format = "PROJ", multiline = "NO"): Discarded datum North_American_Datum_1983 in CRS definition,
##  but +towgs84= values preserved
```

```
plot(GSENM_B, add=T)
```

Above we load the raster into R. We then transform it from one CRS to another so as to match that of the `GSENM_B` shapefile. When changing projections, it is important to remember that `"bilinear"` is best for continous data and `"ngb"` is appropriate for categorical data. Using `epsg` numbers is best for ensuring you are using the correct CRS. The information within the raster is similiar to shapefiles except now we have data on resolution. To plot rasters you can use the base `plot` function in R. To add other layers to the plot, simply use the parameter `add=T`.

# 2 Predictor Variables

Predictor variables are a central component to predictive models. For archaeologcial resource prediction, it is important to pick predictor variables that relate to human land-use patterns. Our predictor variable choices are a product of HBE theory (see main article).

Below is a complete list of the 55 predictors. For more detail see Appendix 1.

```
##  [1] "dem_5m_gsenm"                              
##  [2] "east_west_asp"                             
##  [3] "et_mean_00_13_1"                           
##  [4] "GDD_corngrowing_dds_2005"                  
##  [5] "GDD_doublesine_2005"                       
##  [6] "GDD_doubletriangle_2005"                   
##  [7] "GDD_simpleaverage_2005"                    
##  [8] "GDD_singlesine_2005"                       
##  [9] "GDD_singletriangle_2005"                   
## [10] "Heating_dds_2005"                          
## [11] "lakes_cd"                                  
## [12] "Moisture_Index1"                           
## [13] "north_south_asp"                           
## [14] "NPP_mean_00_15"                            
## [15] "PET_mean_00_13"                            
## [16] "Pred_Fall_ppt"                             
## [17] "Pred_Fall_tdmean"                          
## [18] "Pred_Fall_tmax"                            
## [19] "Pred_Fall_tmean"                           
## [20] "Pred_Fall_tmin"                            
## [21] "Pred_Fall_vpdmax"                          
## [22] "Pred_Fall_vpdmin"                          
## [23] "Pred_Spring_ppt"                           
## [24] "Pred_Spring_tdmean"                        
## [25] "Pred_Spring_tmax"                          
## [26] "Pred_Spring_tmean"                         
## [27] "Pred_Spring_tmin"                          
## [28] "Pred_Spring_vpdmax"                        
## [29] "Pred_Spring_vpdmin"                        
## [30] "Pred_Summer_ppt"                           
## [31] "Pred_Summer_tdmean"                        
## [32] "Pred_Summer_tmax"                          
## [33] "Pred_Summer_tmean"                         
## [34] "Pred_Summer_tmin"                          
## [35] "Pred_Summer_vpdmax"                        
## [36] "Pred_Summer_vpdmin"                        
## [37] "Pred_Winter_ppt"                           
## [38] "Pred_Winter_tdmean"                        
## [39] "Pred_Winter_tmax"                          
## [40] "Pred_Winter_tmean"                         
## [41] "Pred_Winter_tmin"                          
## [42] "Pred_Winter_vpdmax"                        
## [43] "Pred_Winter_vpdmin"                        
## [44] "PRISM_ppt_30yr_normal_800mM2_annual_asc"   
## [45] "PRISM_tdmean_30yr_normal_800mM2_annual_asc"
## [46] "PRISM_tmax_30yr_normal_800mM2_annual_asc"  
## [47] "PRISM_tmean_30yr_normal_800mM2_annual_asc" 
## [48] "PRISM_tmin_30yr_normal_800mM2_annual_asc"  
## [49] "PRISM_vpdmax_30yr_normal_800mM2_annual_asc"
## [50] "PRISM_vpdmin_30yr_normal_800mM2_annual_asc"
## [51] "slope"                                     
## [52] "springs_cd"                                
## [53] "streams_cd"                                
## [54] "wetlands_cd"                               
## [55] "wtrshd_size"
```

## 2.1 Predictor Variable Preparation and Creation

There are many places from which to acquire spatial data for a predictive model. These data often come in a number of formats that are either rasters or shapefiles. Shapefiles cannot be used in a predictive model as shapefiles. Rather, they have to be converted to a raster format first. In addition, many files come in a range of projections and datums. We need to be sure that all data have the same range and projections, else they will not stack.

### 2.1.1 Shapefiles to rasters

Shapefiles come in three common formats: points, lines, and polygons. The shapefiles we have generally fall into two categories: data that represent the distribution of a common attribute in the form of a polygon and data that are features of on the landscape (streams, springs, lakes, etc). How we handle these two types of shapefiles in order to convert them to rasters and use them in our predictive model depends on what we want to learn about human land-use relative to these data.

For data that are polygons that describe a single attribute of the landscape, we need to convert them to a raster. In our study watershed size began as a watershed shapefile polygon. Converting it to a raster involves selecting the cell size and selecting which attribute will be represented as each cell value. The `R` function for converting a polygon to a raster is `raster::rasterize`. This can also be done in ArcMap.

For data on landform features, we are most interested in understanding the distance in time between archaeological sites and the features. While most use euclidian distance, which is probably appropriate in areas with realtively flat topography, we measure distance in time so that we can account for the effects of slope on accessibility. To do this we use Tobler’s Hiking Function and ArcMap to create cost-distance rasters.

### 2.1.2 Resampling Rasters

After you have all the rasters you want to use, I suggest putting them in the same folder. By putting them in the same folder, we can use R to quickly register the filepaths. Depending on the size and number of rasters you have, the process of projecting them to the same CRS, resampling them to the same resolution, and saving the new rasters can be time consuming. Illustrated below is how we did it.

First we get the filepaths of the files within the folder of our predictor data and create an outpath for the file we will use at the end.

```
rlist<-list.files("./GIS/Rasters/DEM_ext/epsg_26912/Continuous", full.names=T)
rlist2<-list.files("./GIS/Rasters/DEM_ext/epsg_26912/Continuous", full.names=F)
#this creates the file paths for where to save the output
outpath<-"D:/Desktop/Grants/CPAA_GSENM/GSENM/Data/GIS/Rasters/FINALDATA/"
outfiles <- paste0(outpath, rlist2)
```

There are many ways to streamline resampling and reprojection. Here, we use a simple `for` loop in which:

The `for` loop:

- loads a raster from the filepath specified in `rlist` and specifies the CRS;
- reprojects the raster into the standard CRS;
- does an initial crop of the raster to the standard extent so as to save time on the resample;
- resamples to the standard resolution specified here by the highest resolution raster;
- crops the output to match the standard output extent of the GSENM\_B;
- saves the raster using the appropriate name and file path specified in the `outfiles` object.
- repeats with the next raster.

```
for (i in (1:length(rlist))){
  x<-raster(rlist[i])
  proj4string(x)<-CRS("+init=epsg:26912")
  x<-projectRaster(from=x, crs=CRS("+init=epsg:26912"), method="bilinear")
  x<-crop(x,DEM)
  x<-resample(x,DEM,"bilinear")
  x<-crop(x,GSENM_B)
  writeRaster(x,filename=outfiles[i], format="GTiff",overwrite=F)
}
```

This is the base code we used, however the processing time can be decreased by vectorizing the code (see `doParallel::foreach`).

### 2.1.3 Generating Seasonal Data

For the predictor data, instead of using the PRISM monthly average data, we convert the monthly average data to seasonal average data. (PRISM data is avaiable through `R` using `prism::get_prism_monthlys()`.)

Below is the code we used to create seasonal data from the monthly data.

We begin by assigning all the PRISM monthly files paths to a list object. We then create a dataframe that indicates all of the rasters we will be creating (seasonal precipitation, temperature difference mean, max temperature, mean temperature, minimum temperature, and vapor pressure deficits) along with the position in the list of the monthly data we will use to create the seasonal data.

```
#Create a list that has the file paths of the monthly data
rlist<-list.files("./PRSIM_Monthly", full.names=T)
#dataframe
d<-data.frame(Season=c(
  "Winter_ppt","Spring_ppt","Summer_ppt","Fall_ppt",
  "Winter_tdmean","Spring_tdmean","Summer_tdmean","Fall_tdmean",
  "Winter_tmax","Spring_tmax","Summer_tmax","Fall_tmax",
  "Winter_tmean","Spring_tmean","Summer_tmean","Fall_tmean",
  "Winter_tmin","Spring_tmin","Summer_tmin","Fall_tmin",
  "Winter_vpdmax","Spring_vpdmax","Summer_vpdmax","Fall_vpdmax",
  "Winter_vpdmin","Spring_vpdmin","Summer_vpdmin","Fall_vpdmin"
),
Start=c(15,18,21,24,28,31,34,37,41,44,47,50,54,57,60,63,67,70,73,76,80,83,86,89,93,96,99,102), 
End=c(17,20,23,26,30,33,36,39,43,46,49,52,56,59,62,65,69,72,75,78,82,85,88,91,95,98,101,104))
```

Next, we find the average values for each of the seasons. We vectorize the process in order to decrease processing time and utilize all the cores present on our computer (minus 1 which we have left for background processes). We do this using `doParallel::foreach`. This can also be performed using a `for` loop.

The code below detects the number of cores present on the machine and sets the parameters for the `foreach` loop. By running `foreach` we are telling R to assign a task to each core of the machine.

Each core is assigned to:

- load the appropriate rasters for a season into a raster stack;
- calculate the mean value of the stack for each cell;
- save the new seasonal raster;
- repeat.

```
library(doParallel)
#set up
cores<-detectCores()
cl<-makeCluster(cores-1)
registerDoParallel(cl)
#vectorized loop
foreach(i=1:nrow(d), .packages="raster") %dopar% {
  x<-raster::stack(rlist[(d[i,2]):(d[i,3])])
  y<-calc(x,mean)
  writeRaster(y,filename=paste0("./Methods_Paper/Predictors/Seasonal/","Pred_",d[i,1]),format="GTiff",overwrite=T)
  }
stopCluster(cl)
```

Once all rasters have been submitted the cluster stops using `stopCluster` and the seasonal data will now be in the Seasonal folder.

## 2.2 Decomposing Aspect

Because aspect is often measured in degrees, which are interpretted by the models as continuous data, we need to convert them to values that R will recognize as either categorical or continous.

Here, we decompose aspect into two continous variables: east aspect and north aspect that range from 1 to -1. In doing so, aspect is represented continously on an east-west plane and a norht-south plane.

To illustrate we create the range of aspect degrees (0-360) and implement the docomposition into `eastish` and `northish`. In `eastish` a -1 value indicates west oriented and a 1 indicates east oriented. In northish a -1 value indicates south and a 1 indicates north oriented.

```
degree_aspect = seq(0,360)
eastish = sin(degree_aspect/180*pi)
northish = cos(degree_aspect/180*pi)
plot(eastish, type='l',ylab="East Aspect")
```

```
plot(northish,type='l',ylab="West Aspect")
```

We can use this same method to decompose the aspect raster into east aspect and north aspect continous variables.

```
east_west_asp<- sin(aspect/180*pi)
north_south_asp<- cos(degree/180*pi)
```

## 2.3 Predictor Variable Refinement

After transforming, resampling, and cropping the rasters so that they are all in the same CRS, have the same resolution, and the same extent, they should be stackable. Now we can look closer at our predictor variables and get an idea of collinearity.

The point of variable selection using PCA is to eliminate strong collinearity in the predictor variable set used in the model. Inclusion of variables with strong collinearity in predictive models will often lead to an overestimate in the predictive power of that model as a result of autocrorrelation within the predictor variables.

Running a PCA in R is pretty simple. Because the rasters used here are so large (6gb each), we only use a sample of cells to run our PCA. Because of this, we run the PCA three times in order to be sure our results are representative. Notice that within the PCA the predictor data values are scaled. For PCA, variables need to be scaled if they are in different units of measurement.

```
#Stack our new Seasonal and all data
rlist<-list.files("./Methods_Paper/Predictors/Seasonal", full.names=T)
predictors<-raster::stack(rlist)
proj4string(predictors)<-CRS("+init=epsg:26912")

#
samp.pca<-prcomp(sampleRandom(predictors,10000,cells=F),scale=T)
save(samp.pca,file="./samp_pca.RData")

#Mrunning it a couple of different times with different samples just to check for stability
samp.pca1<-prcomp(sampleRandom(predictors,10000,cells=F),scale=T)
save(samp.pca1,file="./samp_pca1.RData")

samp.pca2<-prcomp(sampleRandom(predictors,10000,cells=F),scale=T)
save(samp.pca2,file="./samp_pca2.RData")
```

Biplots are the common way of presenting PCA, but since we are concerned with more than just the first two principal components and we have so many observations these can be cluttered. A better way is to sort variables by their eigenvectors (or loadings).

Eigenvector values describe the correlation with each Principal Component. Positive and negative values only relate to the direction of correlation. Therefore, use the absolute value of eigenvectors in order to determine which variables are most representative of each principal component.

We use two criterion for the eigenvectors to decide which variables are best described by each Principal Components that explain up to 95% of the variation. `summary(my.pca)` can be used to determine the proporotion of variance explained by each Principal Component.

The first method is to take the absolute value of eigenvectors and sort them from largest to smallest. Below we only pring the first 10. The code is provided below, but the results are long and not shown.

```
foreach(i=1:10) %do% {sort(abs(samp.pca$rotation[,i]), decreasing=T)}
```

The second method is to use barplots to visually inspect the eigenvectors and their relation to other variables. This is the prefer method. Looking at the barplots, you are able to identify variables that correlate with one another and might be summarized by a single variable, but generally describes a higher dimension quality (i.e. size).

The example shows the eigenvectors of PC1. We can see that PC1 describes mean temperature. From this data, we select the annual mean temperature.

We can also use `kmeans()` to partition our eigenvectors and get an idea of how they cluster.

```
barplot(sort(abs(samp.pca$rotation[,1]),decreasing=T),cex.names=.6, las=2)
```

```
#cluster analsysis to see how variables group and get a sense of what is beign described.
head(kmeans(dist(sort(abs(samp.pca$rotation[,2]), decreasing=T)),centers=10)$cluster)
```

```
##      NPP_mean_00_15     et_mean_00_13_1      PET_mean_00_13         wetlands_cd 
##                   6                   6                   6                  10 
##            lakes_cd GDD_doublesine_2005 
##                  10                   8
```

Alternatively, instead of selecting variables that are described by each PC, you could use the PC itself as the predictor variable. This would require that you convert all predictor variables into principal component space, which may not be possible with large raster datasets. In addition, it would be more difficult to interpret what quality each principal component actually describes.

## 2.4 Predictor Variable Correlation

Because we select variables based on the principal components in the PCA, we expect that there will be some correlation left between the predictor variables. While there is not a solid threshold at which correlation is too great, we still want to see how the refined predictor variables relate to one another.

The greatest correlation (0.42) is between Cost-distance to springs and cost-distance to wetlands.

```
rps_preds<-randomPoints(mask=predictors[[1]],n=20000,ext=GSENM_B)
rps_preds<-data.frame(rps_preds)
rps_preds<-SpatialPointsDataFrame(coords=rps_preds,proj4string=GSENM_B@proj4string,data=rps_preds)
rps_preds<-rps_preds[GSENM_B,]
rps_preds_cor<-extract(predictors,rps_preds)
rps_preds_cor_res<-cor(rps_preds_cor)
```

```
library(corrplot)
```

```
## corrplot 0.84 loaded
```

```
names(predictors)
```

```
##  [1] "east_west_asp"                            
##  [2] "GDD_corngrowing_dds_2005"                 
##  [3] "north_south_asp"                          
##  [4] "NPP_mean_00_15"                           
##  [5] "PRISM_tmean_30yr_normal_800mM2_annual_asc"
##  [6] "slope"                                    
##  [7] "springs_cd"                               
##  [8] "streams_cd"                               
##  [9] "wetlands_cd"                              
## [10] "wtrshd_size"
```

```
corrplot.mixed(rps_preds_cor_res,lower.col = "black", number.cex = .7,tl.pos="n")
```

```
#rps_preds_cor_res
```

## 2.5 Representative Sample Test

One of the major criqitues about archaeological predictive models is that they often fail to get a representative sample. Some interpret this as having to do with a sample of sites, but in reality it is an appropriate sample of the landscape. In order for our sample to be representative we need to be sure that we have collected data (both presence and absence) across the range of potential environments (combinations of raster cell values) that exist. Getting a representative sample can be complicated for reasons outlined in our paper and elsewhere. Here we focus on ways of addressing whether a sample is representative.

Here, we use only the refined rasters because of the processing power and time required to adequately address the range of values in the rasters. We usea mean difference approach in which we subtract the sample mean from the extent mean.

To test whether the sampled area (areas where inventories have occurred) is representative of the range of possible habitats, we use a permutation test that uses a distribution of mean-differences on a series of sample points from the predictors within and outside (extent) of the sampled area. We draw a random sample from the sampled area and the extent 100 times for each predictor variable and calculate the difference of means to obtain a distribution of mean-differences. The mean of the mean-differences distributions will have a mean close to 0 if the sampled area is representative of the range of possible habitats. If our sample falls within 95% of the extent distribution, we conclude that it is representative.

The Mean difference approach is not sensitive to differences in the distribution. Since we are using a sample, we repeat the test 100 times for each predictor variable.

To calculate the mean and SD differences we:

- create a dataframe to store all measuresments (`mean_sd_dif`);
- generate random points within the inventoried areas;
- calculate the mean value of predictors within the inventoried area;
- caculate the mean value of a sample of predictors across the extent;
- caculate the difference between the extent mean and inventoried mean;
- add it to our table;
- calculate the SD value of predictors within the inventoried area;
- caculate the SD value of a sample of predictors across the extent;
- caculate the difference between the extent SD and inventoried SD;
- add it to our table;
- repeat.

```
mean_sd_dif<-data.frame(predictor=1:10, mean_dif=1:1000, SD_dif=1:1000)

for (i in (1:nrow(mean_sd_dif))){
  rps<-randomPoints(mask=predictors[[1]],n=1000,ext=GSENM_B)
  rps<-data.frame(rps)
  rps<-SpatialPointsDataFrame(coords=rps,proj4string=GSENM_B@proj4string,data=rps)
  #cropping action
  rps<-rps[inventories,]
  
  samp_mean<-mean(extract(predictors[[mean_sd_dif[i,1]]],rps))
  pop_mean<-mean(sampleRandom(predictors[[mean_sd_dif[i,1]]],10000,cells=F))
  
  mean_sd_dif[i,2]<-pop_mean - samp_mean
  
  samp_sd<-sd(extract(predictors[[mean_sd_dif[i,1]]],rps))
  pop_sd<-sd(sampleRandom(predictors[[mean_sd_dif[i,1]]],10000,cells=F))
  
  mean_sd_dif[i,3]<-pop_sd - samp_sd
}
```

We can visualize the results using a histogram. A mean difference close to 0 suggests a representative sample, whereas a mean difference greater than 0 indicates higher values found at the extent level, and a mean difference less than 0 indicates higher values found at the inventory level.

We can determine where the inventoried cell values fall in the distribution of extent values to estimate a level of of significance at 0.05 using the `pnorm()` function.

```
par(mfrow=c(4,3))
rep_test<-data.frame(pred_num=c(1:10),pred_name=names(predictors),p_val=NA)
#hist(mean_sd_dif[mean_sd_dif$predictor==1,]$mean_dif, main="East-West Aspect")
rep_test[1,3]<-pnorm(0,mean=mean(mean_sd_dif[mean_sd_dif$predictor==1,]$mean_dif),sd=sd(mean_sd_dif[mean_sd_dif$predictor==1,]$mean_dif))


#hist(mean_sd_dif[mean_sd_dif$predictor==2,]$mean_dif, main= "GDD")
rep_test[2,3]<-pnorm(0,mean=mean(mean_sd_dif[mean_sd_dif$predictor==2,]$mean_dif),sd=sd(mean_sd_dif[mean_sd_dif$predictor==2,]$mean_dif))


#hist(mean_sd_dif[mean_sd_dif$predictor==3,]$mean_dif, main= "North-South Aspect")
rep_test[3,3]<-pnorm(0,mean=mean(mean_sd_dif[mean_sd_dif$predictor==3,]$mean_dif),sd=sd(mean_sd_dif[mean_sd_dif$predictor==3,]$mean_dif))


#hist(mean_sd_dif[mean_sd_dif$predictor==4,]$mean_dif, main="NPP")
rep_test[4,3]<-pnorm(0,mean=mean(mean_sd_dif[mean_sd_dif$predictor==4,]$mean_dif),sd=sd(mean_sd_dif[mean_sd_dif$predictor==4,]$mean_dif))


#hist(mean_sd_dif[mean_sd_dif$predictor==5,]$mean_dif, main="Mean Temp")
rep_test[5,3]<-pnorm(0,mean=mean(mean_sd_dif[mean_sd_dif$predictor==5,]$mean_dif),sd=sd(mean_sd_dif[mean_sd_dif$predictor==5,]$mean_dif))


#hist(mean_sd_dif[mean_sd_dif$predictor==6,]$mean_dif,main="Slope")
rep_test[6,3]<-pnorm(0,mean=mean(mean_sd_dif[mean_sd_dif$predictor==6,]$mean_dif),sd=sd(mean_sd_dif[mean_sd_dif$predictor==6,]$mean_dif))


#hist(mean_sd_dif[mean_sd_dif$predictor==7,]$mean_dif,main="Cost-Distance to Springs")
rep_test[7,3]<-pnorm(0,mean=mean(mean_sd_dif[mean_sd_dif$predictor==7,]$mean_dif),sd=sd(mean_sd_dif[mean_sd_dif$predictor==7,]$mean_dif))


#hist(mean_sd_dif[mean_sd_dif$predictor==8,]$mean_dif,main="Cost-Distance to Streams")
rep_test[8,3]<-pnorm(0,mean=mean(mean_sd_dif[mean_sd_dif$predictor==8,]$mean_dif),sd=sd(mean_sd_dif[mean_sd_dif$predictor==8,]$mean_dif))


#hist(mean_sd_dif[mean_sd_dif$predictor==9,]$mean_dif,main="Cost-Distance to Wetlands")
rep_test[9,3]<-pnorm(0,mean=mean(mean_sd_dif[mean_sd_dif$predictor==9,]$mean_dif),sd=sd(mean_sd_dif[mean_sd_dif$predictor==9,]$mean_dif))


#hist(mean_sd_dif[mean_sd_dif$predictor==10,]$mean_dif, main="Watershed Size")
rep_test[10,3]<-pnorm(0,mean=mean(mean_sd_dif[mean_sd_dif$predictor==10,]$mean_dif),sd=sd(mean_sd_dif[mean_sd_dif$predictor==10,]$mean_dif))

rep_test[,3]<-round(rep_test[,3],5)

rep_test
```

```
##    pred_num                                 pred_name   p_val
## 1         1                             east_west_asp 0.64852
## 2         2                  GDD_corngrowing_dds_2005 0.63700
## 3         3                           north_south_asp 0.27928
## 4         4                            NPP_mean_00_15 0.00744
## 5         5 PRISM_tmean_30yr_normal_800mM2_annual_asc 0.08300
## 6         6                                     slope 0.00410
## 7         7                                springs_cd 0.29579
## 8         8                                streams_cd 0.00681
## 9         9                               wetlands_cd 0.00238
## 10       10                               wtrshd_size 0.00000
```

```
par(mfrow=c(1,1))
```

The table above shows that we have a representative sample (p>0.05) for 5 of our predictor variables. This is unfortunate, but we can make sense of many of the non-representative variables based on what we know about archaeological inventory. Slope is the most obvious. We bias inventories to flat areas. The analysis also shows a bias towards sampling closer to springs, streams, and wetlands and in smaller watersheds. The bias sample of NPP suggests invetories focus on higher NPP areas. This is potentially a product of the proximity to water sources.

Even though 5 of our predictor variables have bias inventory data, we will still use them. A more cautious approach would be to exclude these 5 variables from our models, but we believe exclusion would detract more from our models than inclusion with the knowledge that some variables might introduce biases in the predictions.

# 3 Response Variable

After Identifying the set of predictor variables, we now need to get our presence (archaeological) data and absence data prepped and standardized.

*We use Formative Period archaeological sties from the Grand Staircase-Escalante National Monument for our presence data. Archaeological data has restricted access and therefore do not provide archaeological site location data.*

First we need to load the necessary data. We need the selection of predictor variables and a project boundary. The project boundary should be the extent to which presence points have been looked for.

```
rlist<-list.files("./Methods_Paper/Predictors/Final", full.names=T)
predictors<-raster::stack(rlist)
proj4string(predictors)<-CRS("+init=epsg:26912")
#boundary
GSENM_B<-readShapePoly('./Data/GIS/Shapefiles/GSENM_B/GSENM_boundary') #shapefile of the state
proj4string(GSENM_B)<-CRS("+init=epsg:26912")
```

## 3.1 Presence Data

Next we will load our presence data. Below we load the site data (spatial data not provided) and subset it so that we only have Formative Period sites (FO = unknown formative, PB = Ancestral Puebloan, FR = Fremont) and subset these so that we only have Formative Period sites within the GSENM boundary. Definitions of these site types are found in Yaworsky et al. 2018 (see main article).

The site data are composed of 1,619 Formative Period sites on the GSENM. We reduce spatial data to the lowest common denominator, point data. For polygon data, we use the centroid of the polygon so that all data is in the form of spatial points.

```
load("../all_sites_final.Rda")
pres<-subset(all_sites,TIME =="FO"| TIME=="PB" | TIME=="FR")
pres<-pres[GSENM_B,]
rm(all_sites)
```

## 3.2 Absence Data

Next, we can generate our absence points. In reality they are not true absence points, but rather psuedo-absence points or background points. They allow the models to get an idea of the range of habitats that exist. As discussed in the main paper, many modeling approaches and measures of predictive power assume true-absence points.

Absence points are generated within the extent of the GSENM. We begin by generating 20,000 points and then remove points outside of the GSENM boundary resulting in a total of 10,195 absence points. The points are randomly generated but avoid raster cells with known presence points. Our target number is at least 10,000 points within the GSENM boundary (Barbet-Massin et al., 2012)

```
abs<-randomPoints(mask=predictors[[1]],n=20000,p=pres,ext=GSENM_B)
abs<-data.frame(abs)
abs<-SpatialPointsDataFrame(coords=abs,proj4string=GSENM_B@proj4string,data=abs)
#cropping action
abs<-abs[GSENM_B,]
```

## 3.3 Extracting Predictor Data

Next, we extract the predictor variables values of each presence and absence point and combine them into a single dataframe. 0 will represent absence and 1 will represent presence.

```
pres_vals<-extract(predictors,pres)
abs_vals<-extract(predictors,abs)
pa<- c(rep(1, nrow(pres_vals)), rep(0, nrow(abs_vals)))
sdmdata <- data.frame(cbind(pa, rbind(pres_vals, abs_vals)))
sdmdata<-na.omit(sdmdata)
head(sdmdata)
```

Here we only use the Formative Period sites, but we have provided the data necessary for others to look into predicting Formative Period land use.

# 4 Model Setups

One of the primary goals of the paper is to address the statistical approaches and determine the pros and cons of the available statistical approaches. To adequately assess the different modeling approaches we need to be sure that we are using the same data in each. To do this we partition our presence and absence data into four folds. The four folds will allow us to use the same data for training and testing each modeling approach in a four fold cross-validation.

```
train_group <- kfold(sdmdata, 4) #splitting into 4 groups
```

Below we are creating a dataframe in which to record model results (AUC and Optimal Threshold values).

```
All_Results<-data.frame(Run=1:4, stepGLM_AUC=4,GLM_AUC=4,GAM_AUC=4,ME_AUC=4,RF_AUC=4,XG_AUC=4, stepGLM_Thres=4,GLM_Thres=4,GAM_Thres=4,ME_Thres=4,RF_Thres=4, XG_Thres=4)
```

## 4.1 GLM Stepwise setup

In the paper we use a GLM without conducting a stepwise variable selection pre-analysis. This is because we wanted to maintain data consistency across all modeling approaches. Here, we generate a stepwise GLM in which we have used a stepwise variable selection to minimize the AIC score. The stepwise variable selection will give us a model with the lowest AIC.

We conduct the stepwise variable selection using the first k-fold of data.

```
#tuning the GLM
train <- sdmdata[train_group != All_Results[1,1], ] #model training gropu, not equal to i
pres_train<-train[train$pa==1,] #all the presence points in the training group
abs_train<-train[train$pa==0,] # all the absence poings in the training group
test <- sdmdata[train_group == All_Results[1,1], ] #model test gropu, equal to i
pres_test<-test[test$pa==1,] #all presence in test data
abs_test<-test[test$pa==0,] #all absence in test data
Full.glm<-glm(pa ~ .,family = binomial(link = "logit"), data=train)
summary(Full.glm)

#stepwise model selection using AIC
null.glm <- glm(pa ~ 1,family=binomial,data=train)
step(null.glm, scope=formula(Full.glm))
```

From the stepwise variable selection we are able to identify the best glm setup based on AIC (see model setup below). The full model has an AIC of 5960 and the stepwise variable selection identifies 7 variables that give an AIC of 5956. A minor improvement.

```
step(null.glm, scope=formula(Full.glm))
```

```
## Start:  AIC=6977.64
## pa ~ 1
## 
##                                             Df Deviance    AIC
## + springs_cd                                 1   6520.5 6524.5
## + wetlands_cd                                1   6572.2 6576.2
## + GDD_corngrowing_dds_2005                   1   6743.2 6747.2
## + wtrshd_size                                1   6810.3 6814.3
## + slope                                      1   6917.8 6921.8
## + north_south_asp                            1   6943.0 6947.0
## + east_west_asp                              1   6965.1 6969.1
## <none>                                           6975.6 6977.6
## + NPP_mean_00_15                             1   6974.0 6978.0
## + streams_cd                                 1   6974.5 6978.5
## + PRISM_tmean_30yr_normal_800mM2_annual_asc  1   6974.8 6978.8
## 
## Step:  AIC=6524.55
## pa ~ springs_cd
## 
##                                             Df Deviance    AIC
## + GDD_corngrowing_dds_2005                   1   6302.7 6308.7
## + wetlands_cd                                1   6362.6 6368.6
## + wtrshd_size                                1   6363.8 6369.8
## + north_south_asp                            1   6488.8 6494.8
## + slope                                      1   6501.7 6507.7
## + east_west_asp                              1   6512.7 6518.7
## <none>                                           6520.5 6524.5
## + PRISM_tmean_30yr_normal_800mM2_annual_asc  1   6520.5 6526.5
## + streams_cd                                 1   6520.5 6526.5
## + NPP_mean_00_15                             1   6520.5 6526.5
## - springs_cd                                 1   6975.6 6977.6
## 
## Step:  AIC=6308.69
## pa ~ springs_cd + GDD_corngrowing_dds_2005
## 
##                                             Df Deviance    AIC
## + wtrshd_size                                1   6135.2 6143.2
## + wetlands_cd                                1   6151.0 6159.0
## + PRISM_tmean_30yr_normal_800mM2_annual_asc  1   6229.4 6237.4
## + north_south_asp                            1   6271.2 6279.2
## + slope                                      1   6295.0 6303.0
## + east_west_asp                              1   6297.0 6305.0
## <none>                                           6302.7 6308.7
## + NPP_mean_00_15                             1   6301.7 6309.7
## + streams_cd                                 1   6302.3 6310.3
## - GDD_corngrowing_dds_2005                   1   6520.5 6524.5
## - springs_cd                                 1   6743.2 6747.2
## 
## Step:  AIC=6143.24
## pa ~ springs_cd + GDD_corngrowing_dds_2005 + wtrshd_size
## 
##                                             Df Deviance    AIC
## + wetlands_cd                                1   5989.7 5999.7
## + north_south_asp                            1   6105.8 6115.8
## + PRISM_tmean_30yr_normal_800mM2_annual_asc  1   6120.7 6130.7
## + east_west_asp                              1   6127.1 6137.1
## + slope                                      1   6130.9 6140.9
## <none>                                           6135.2 6143.2
## + streams_cd                                 1   6134.0 6144.0
## + NPP_mean_00_15                             1   6135.2 6145.2
## - wtrshd_size                                1   6302.7 6308.7
## - GDD_corngrowing_dds_2005                   1   6363.8 6369.8
## - springs_cd                                 1   6566.9 6572.9
## 
## Step:  AIC=5999.75
## pa ~ springs_cd + GDD_corngrowing_dds_2005 + wtrshd_size + wetlands_cd
## 
##                                             Df Deviance    AIC
## + north_south_asp                            1   5957.3 5969.3
## + PRISM_tmean_30yr_normal_800mM2_annual_asc  1   5977.5 5989.5
## + east_west_asp                              1   5983.0 5995.0
## <none>                                           5989.7 5999.7
## + streams_cd                                 1   5988.9 6000.9
## + NPP_mean_00_15                             1   5989.4 6001.4
## + slope                                      1   5989.5 6001.5
## - wetlands_cd                                1   6135.2 6143.2
## - wtrshd_size                                1   6151.0 6159.0
## - GDD_corngrowing_dds_2005                   1   6210.6 6218.6
## - springs_cd                                 1   6228.9 6236.9
## 
## Step:  AIC=5969.35
## pa ~ springs_cd + GDD_corngrowing_dds_2005 + wtrshd_size + wetlands_cd + 
##     north_south_asp
## 
##                                             Df Deviance    AIC
## + PRISM_tmean_30yr_normal_800mM2_annual_asc  1   5945.1 5959.1
## + east_west_asp                              1   5951.6 5965.6
## <none>                                           5957.3 5969.3
## + streams_cd                                 1   5955.6 5969.6
## + NPP_mean_00_15                             1   5957.1 5971.1
## + slope                                      1   5957.2 5971.2
## - north_south_asp                            1   5989.7 5999.7
## - wetlands_cd                                1   6105.8 6115.8
## - wtrshd_size                                1   6115.7 6125.7
## - GDD_corngrowing_dds_2005                   1   6178.1 6188.1
## - springs_cd                                 1   6193.2 6203.2
## 
## Step:  AIC=5959.1
## pa ~ springs_cd + GDD_corngrowing_dds_2005 + wtrshd_size + wetlands_cd + 
##     north_south_asp + PRISM_tmean_30yr_normal_800mM2_annual_asc
## 
##                                             Df Deviance    AIC
## + east_west_asp                              1   5939.6 5955.6
## <none>                                           5945.1 5959.1
## + streams_cd                                 1   5943.7 5959.7
## + NPP_mean_00_15                             1   5944.8 5960.8
## + slope                                      1   5945.0 5961.0
## - PRISM_tmean_30yr_normal_800mM2_annual_asc  1   5957.3 5969.3
## - north_south_asp                            1   5977.5 5989.5
## - wtrshd_size                                1   6051.9 6063.9
## - wetlands_cd                                1   6091.5 6103.5
## - GDD_corngrowing_dds_2005                   1   6166.6 6178.6
## - springs_cd                                 1   6174.8 6186.8
## 
## Step:  AIC=5955.6
## pa ~ springs_cd + GDD_corngrowing_dds_2005 + wtrshd_size + wetlands_cd + 
##     north_south_asp + PRISM_tmean_30yr_normal_800mM2_annual_asc + 
##     east_west_asp
## 
##                                             Df Deviance    AIC
## <none>                                           5939.6 5955.6
## + streams_cd                                 1   5938.2 5956.2
## + NPP_mean_00_15                             1   5939.3 5957.3
## + slope                                      1   5939.5 5957.5
## - east_west_asp                              1   5945.1 5959.1
## - PRISM_tmean_30yr_normal_800mM2_annual_asc  1   5951.6 5965.6
## - north_south_asp                            1   5971.0 5985.0
## - wtrshd_size                                1   6047.8 6061.8
## - wetlands_cd                                1   6084.9 6098.9
## - GDD_corngrowing_dds_2005                   1   6159.4 6173.4
## - springs_cd                                 1   6168.2 6182.2
```

```
## 
## Call:  glm(formula = pa ~ springs_cd + GDD_corngrowing_dds_2005 + wtrshd_size + 
##     wetlands_cd + north_south_asp + PRISM_tmean_30yr_normal_800mM2_annual_asc + 
##     east_west_asp, family = binomial, data = train)
## 
## Coefficients:
##                               (Intercept)  
##                                -8.192e-01  
##                                springs_cd  
##                                -1.405e-02  
##                  GDD_corngrowing_dds_2005  
##                                 9.136e-04  
##                               wtrshd_size  
##                                -1.239e-08  
##                               wetlands_cd  
##                                -2.009e-02  
##                           north_south_asp  
##                                -2.674e-01  
## PRISM_tmean_30yr_normal_800mM2_annual_asc  
##                                -9.780e-02  
##                             east_west_asp  
##                                 1.113e-01  
## 
## Degrees of Freedom: 8859 Total (i.e. Null);  8852 Residual
## Null Deviance:       6976 
## Residual Deviance: 5940  AIC: 5956
```

## 4.2 Model Construction and Runs

Since we are using a four fold cross validation, we will run each of the four models four times. Each fold will use 75% of the data to train the model and the omited 25% to test the model. Essentially we are assessing the models predictive power by seeing how well it can predict the omited test data. All model results are included in the RData file. Model evaluation results (AUC and threshold at max TPR+TNR) are found in `All_Results`.

We will walk you through the first k fold. The additional k folds follow the same pattern, but use different partitions of the data for training and testing as part of the cross validation.

Note that implementing the code used here may result in slightly different results as a result of the use of different data partitions and different background points. That said, results should be very similiar to ours assuming the random sample and the data partitions are representative.

# 5 Model Runs

## 5.1 K-fold 1 setup

```
#Kfold 1 and all models 1!
train <- sdmdata[train_group != All_Results[1,1], ] #model training gropu, not equal to i
pres_train<-train[train$pa==1,] #all the presence points in the training group
abs_train<-train[train$pa==0,] # all the absence poings in the training group
test <- sdmdata[train_group == All_Results[1,1], ] #model test gropu, equal to i
pres_test<-test[test$pa==1,] #all presence in test data
abs_test<-test[test$pa==0,] #all absence in test data
```

### 5.1.1 K-fold 1 GLMs

The stepGLM setup uses only the variables identified in the stepwise variable selection above. The model results for Kfold 1 stepGLM are found in `FR.stepGLM_1`. In the script below, we create a stepGLM using the training data. Next we evaluate the models performance using AUC and determine an optimal threshold level (see paper on threshold at max TPR+TNR).

Next, we create the GLM with all 10 variables. The model results are found in`FR.glm_1`.

```
FR.stepglm_1<-glm(as.factor(pa) ~ 
              north_south_asp
              + east_west_asp 
              +GDD_corngrowing_dds_2005
              +PRISM_tmean_30yr_normal_800mM2_annual_asc
              +springs_cd
              +wetlands_cd
              +wtrshd_size
              ,family = binomial(link = "logit"), data=train)

All_Results[1,8]<-threshold(evaluate(pres_test, abs_test, FR.stepglm_1))[2] #this is also done within evaluate
All_Results[1,2]<-evaluate(pres_test, abs_test, FR.stepglm_1)@auc

#Full model
FR.glm_1<-glm(as.factor(pa) ~.,family = binomial(link = "logit"), data=train)
All_Results[1,9]<-threshold(evaluate(pres_test, abs_test, FR.glm_1))[2] #this is also done within evaluate
All_Results[1,3]<-evaluate(pres_test, abs_test, FR.glm_1)@auc
```

### 5.1.2 K-fold 1 GAM

The GAM set up is similiar to the GLM set up except that we use all of the variables identified in the PCA. By setting the parameter `select=T` we tell the model to automatically weight predictor variable contribution to the model. In addition, the GAM allows for nonlinear response curves between variables, meaning that our model should have a better fit to our data. Again, the GAM below is created using the training data of kfold 1 and the AUC and threshold values are saved in a table (`All_Results`). The model results for kfold 1 GAM are found in `FR.GAM_1`.

```
#GAM
#not sure how to properly set this up
FR.gam_1<-gam(as.factor(pa) ~ s(north_south_asp)+
                s(east_west_asp)+
                s(GDD_corngrowing_dds_2005)+
                s(NPP_mean_00_15)+
                s(PRISM_tmean_30yr_normal_800mM2_annual_asc)+
                s(slope)+
                s(springs_cd)+
                s(streams_cd)+
                s(wetlands_cd)+
                s(wtrshd_size)
              ,family = binomial(link = "logit"), data=train, select=T)
All_Results[1,10]<-threshold(evaluate(pres_test, abs_test, FR.gam_1))[2]
All_Results[1,4]<-evaluate(pres_test, abs_test, FR.gam_1)@auc
```

### 5.1.3 K-fold 1 MaxEnt

The MaxEnt approach has a slightly different set up than the GLM and GAM. The standard MaxEnt set up allows users to input the predictor data stack, presence points, and have maxent generate background points. In order to maintain data consistency in our modeling efforts, we have already extracted the values of our predictor data. `maxent_train` is a dataframe with each column being the values of predictor variables and each row being a presence or absence point. `me_pa` is then a dataframe of 1 and 0 indicating presence or absence and corresponds with the rows in `maxent_train`. Maxent results are found in the supplementary material folder of FR.Maxent.

```
#maxent
maxent_train<-data.frame(rbind(pres_train,abs_train))
me_pa<-maxent_train[,1]
maxent_train<-maxent_train[,-1]
jar <- paste(system.file(package="dismo"), "/java/maxent.jar", sep='')
if(file.exists(jar)){
  FR.maxent_1<-maxent(x=maxent_train,p=as.factor(me_pa),path="./FR.MaxEnt/1")
}
#evaluate(pres_test, abs_test, FR.maxent)
All_Results[1,5]<-evaluate(pres_test, abs_test, FR.maxent_1)@auc
All_Results[1,11]<-threshold(evaluate(pres_test, abs_test, FR.maxent_1))[2]
```

### 5.1.4 K-fold 1 Random Forest

The Random Forest approach has a very simple setup in which we create the model (`model` which looks at the relationship between presence and absence (`pa`) and the predictor variables (`.`). By specifying the parameter `importance=T` we are telling the model to evaluate predictor variable importance. This is done using `%IncMSE`, which measures the increase in mse of predictions (estimated with out-of-bag-CV) as a result of permuting individual predictor variables. The Random Forest approach below uses a regression method in which it perceives `pa` as continous data rather than a factor. We could specify `pa` as a factor and Random Forest would use a classificatory method for prediction, but others have found better results with the regression method in SDM applications (see paper).

```
#Random Forest
model <- pa ~ .
FR.randfor_1 <- randomForest(model, data=na.omit(train), importance=T)
#evaluate(pres_test, abs_test, FR.randfor)
All_Results[1,6]<-evaluate(pres_test, abs_test, FR.randfor_1)@auc
All_Results[1,12]<-threshold(evaluate(pres_test, abs_test, FR.randfor_1))[2]
```

### 5.1.5 K-fold 1 Xtreme Gradient Boosting

In addition to the 4 modeling approaches presented in the paper, we also implemented a newer machine learning regression tree type analysis called Xtreme Gradiant Boosting. The model set up is a bit more cumbersome and the parameter settings withing the model are still dificult to understand. We did not include this method in the paper because of the complexity, it’s novelity, and because it did not perform any better than Random Forest.

```
## Setup xgboost data structures
## This first line converts categorical variables to proper dummy variables
train.x <- sparse.model.matrix(pa~.-1, data = train)
train.y = train[,"pa"]
test.x <- sparse.model.matrix(pa~.-1, data = test)
test.y = test[,"pa"]
## Run xgboost
## max.depth: size of trees
## eta: gradient step
## nthread: cpu threads used
## nrounds: number of iterations (increase this if eta is decreased)
## objective: type of training algorithm
FR.bst_1 <- xgboost(data = train.x, label = train.y, max_depth = 4,
                    eta = 0.25, nthread = 2, nrounds=500,objective = "binary:logistic")
fit.pred <- predict(FR.bst_1, newdata=test.x)
fit.ev <- evaluate(p=fit.pred[test.y==1], a=fit.pred[test.y==0])
All_Results[1,7] <- fit.ev@auc
All_Results[1,13] <- threshold(fit.ev)$spec_sens
```

## 5.2 K-folds 2-4

Folds 2 through 4 are implemented the same way as fold 1, but with rotating training and test data. These model outputs can be called from the RData file.

```
#kfold 2
train <- sdmdata[train_group != All_Results[2,1], ] #model training gropu, not equal to i
pres_train<-train[train$pa==1,] #all the presence points in the training group
abs_train<-train[train$pa==0,] # all the absence poings in the training group
test <- sdmdata[train_group == All_Results[2,1], ] #model test gropu, equal to i
pres_test<-test[test$pa==1,] #all presence in test data
abs_test<-test[test$pa==0,] #all absence in test data

FR.stepglm_2<-glm(as.factor(pa) ~ 
              north_south_asp
              + east_west_asp 
              +GDD_corngrowing_dds_2005
              +PRISM_tmean_30yr_normal_800mM2_annual_asc
              +springs_cd
              +wetlands_cd
              +wtrshd_size
              ,family = binomial(link = "logit"), data=train)
All_Results[2,8]<-threshold(evaluate(pres_test, abs_test, FR.stepglm_2))[2] #this is also done within evaluate
All_Results[2,2]<-evaluate(pres_test, abs_test, FR.stepglm_2)@auc

FR.glm_2<-glm(as.factor(pa) ~.,family = binomial(link = "logit"), data=train)
All_Results[2,9]<-threshold(evaluate(pres_test, abs_test, FR.glm_2))[2] #this is also done within evaluate
All_Results[2,3]<-evaluate(pres_test, abs_test, FR.glm_2)@auc

#GAM
#not sure how to properly set this up
FR.gam_2<-gam(as.factor(pa) ~ s(east_west_asp)+
                s(north_south_asp)+
                s(GDD_corngrowing_dds_2005)+
                s(NPP_mean_00_15)+
                s(PRISM_tmean_30yr_normal_800mM2_annual_asc)+
                s(slope)+
                s(springs_cd)+
                s(streams_cd)+
                s(wetlands_cd)+
                s(wtrshd_size)
              ,family = binomial(link = "logit"), data=train, select=T)
All_Results[2,10]<-threshold(evaluate(pres_test, abs_test, FR.gam_2))[2]
All_Results[2,4]<-evaluate(pres_test, abs_test, FR.gam_2)@auc


maxent_train<-data.frame(rbind(pres_train,abs_train))
me_pa<-maxent_train[,1]
maxent_train<-maxent_train[,-1]
jar <- paste(system.file(package="dismo"), "/java/maxent.jar", sep='')
if(file.exists(jar)){
  FR.maxent_2<-maxent(x=maxent_train,p=as.factor(me_pa),path="./FR.MaxEnt/2")
}
#evaluate(pres_test, abs_test, FR.maxent)
All_Results[2,5]<-evaluate(pres_test, abs_test, FR.maxent_2)@auc
All_Results[2,11]<-threshold(evaluate(pres_test, abs_test, FR.maxent_2))[2]

#Random Forest
model <- pa ~ .
FR.randfor_2 <- randomForest(model, data=na.omit(train),importance=T)
#evaluate(pres_test, abs_test, FR.randfor)
All_Results[2,6]<-evaluate(pres_test, abs_test, FR.randfor_2)@auc
All_Results[2,12]<-threshold(evaluate(pres_test, abs_test, FR.randfor_2))[2]
#Hyper Volumes
## Setup xgboost data structures
## This first line converts categorical variables to proper dummy variables
train.x <- sparse.model.matrix(pa~.-1, data = train)
train.y = train[,"pa"]
test.x <- sparse.model.matrix(pa~.-1, data = test)
test.y = test[,"pa"]
## Run xgboost
## max.depth: size of trees
## eta: gradient step
## nthread: cpu threads used
## nrounds: number of iterations (increase this if eta is decreased)
## objective: type of training algorithm
FR.bst_2 <- xgboost(data = train.x, label = train.y, max_depth = 4,
                    eta = 0.25, nthread = 2, nrounds=500,objective = "binary:logistic")
fit.pred <- predict(FR.bst_2, newdata=test.x)
fit.ev <- evaluate(p=fit.pred[test.y==1], a=fit.pred[test.y==0])
All_Results[2,7] <- fit.ev@auc
All_Results[2,13] <- threshold(fit.ev)$spec_sens

#############################################################################################
#kfold 3
train <- sdmdata[train_group != All_Results[3,1], ] #model training gropu, not equal to i
pres_train<-train[train$pa==1,] #all the presence points in the training group
abs_train<-train[train$pa==0,] # all the absence poings in the training group
test <- sdmdata[train_group == All_Results[3,1], ] #model test gropu, equal to i
pres_test<-test[test$pa==1,] #all presence in test data
abs_test<-test[test$pa==0,] #all absence in test data

FR.stepglm_3<-glm(as.factor(pa) ~ 
              north_south_asp
              + east_west_asp 
              +GDD_corngrowing_dds_2005
              +PRISM_tmean_30yr_normal_800mM2_annual_asc
              +springs_cd
              +wetlands_cd
              +wtrshd_size
              ,family = binomial(link = "logit"), data=train)
All_Results[3,8]<-threshold(evaluate(pres_test, abs_test, FR.stepglm_3))[2] #this is also done within evaluate
All_Results[3,2]<-evaluate(pres_test, abs_test, FR.stepglm_3)@auc

FR.glm_3<-glm(as.factor(pa) ~.,family = binomial(link = "logit"), data=train)
All_Results[3,9]<-threshold(evaluate(pres_test, abs_test, FR.glm_3))[2] #this is also done within evaluate
All_Results[3,3]<-evaluate(pres_test, abs_test, FR.glm_3)@auc

#GAM
#not sure how to properly set this up
FR.gam_3<-gam(as.factor(pa) ~ s(east_west_asp)+
                s(north_south_asp)+
                s(GDD_corngrowing_dds_2005)+
                s(NPP_mean_00_15)+
                s(PRISM_tmean_30yr_normal_800mM2_annual_asc)+
                s(slope)+
                s(springs_cd)+
                s(streams_cd)+
                s(wetlands_cd)+
                s(wtrshd_size)
              ,family = binomial(link = "logit"), data=train, select=T)
All_Results[3,10]<-threshold(evaluate(pres_test, abs_test, FR.gam_3))[2]
All_Results[3,4]<-evaluate(pres_test, abs_test, FR.gam_3)@auc

maxent_train<-data.frame(rbind(pres_train,abs_train))
me_pa<-maxent_train[,1]
maxent_train<-maxent_train[,-1]
jar <- paste(system.file(package="dismo"), "/java/maxent.jar", sep='')
if(file.exists(jar)){
  FR.maxent_3<-maxent(x=maxent_train,p=as.factor(me_pa),path="./FR.MaxEnt/3")
}
#evaluate(pres_test, abs_test, FR.maxent)
All_Results[3,5]<-evaluate(pres_test, abs_test, FR.maxent_3)@auc
All_Results[3,11]<-threshold(evaluate(pres_test, abs_test, FR.maxent_3))[2]

#Random Forest
model <- pa ~ .
FR.randfor_3 <- randomForest(model, data=na.omit(train),importance=T)
#evaluate(pres_test, abs_test, FR.randfor)
All_Results[3,6]<-evaluate(pres_test, abs_test, FR.randfor_3)@auc
All_Results[3,12]<-threshold(evaluate(pres_test, abs_test, FR.randfor_3))[2]

#Hyper Volumes
## Setup xgboost data structures
## This first line converts categorical variables to proper dummy variables
train.x <- sparse.model.matrix(pa~.-1, data = train)
train.y = train[,"pa"]
test.x <- sparse.model.matrix(pa~.-1, data = test)
test.y = test[,"pa"]
## Run xgboost
## max.depth: size of trees
## eta: gradient step
## nthread: cpu threads used
## nrounds: number of iterations (increase this if eta is decreased)
## objective: type of training algorithm
FR.bst_3 <- xgboost(data = train.x, label = train.y, max_depth = 4,
                    eta = 0.25, nthread = 2, nrounds=500,objective = "binary:logistic")
fit.pred <- predict(FR.bst_3, newdata=test.x)
fit.ev <- evaluate(p=fit.pred[test.y==1], a=fit.pred[test.y==0])
All_Results[3,7] <- fit.ev@auc
All_Results[3,13] <- threshold(fit.ev)$spec_sens

############################################################
#kfold 4
train <- sdmdata[train_group != All_Results[4,1], ] #model training gropu, not equal to i
pres_train<-train[train$pa==1,] #all the presence points in the training group
abs_train<-train[train$pa==0,] # all the absence poings in the training group
test <- sdmdata[train_group == All_Results[4,1], ] #model test gropu, equal to i
pres_test<-test[test$pa==1,] #all presence in test data
abs_test<-test[test$pa==0,] #all absence in test data

FR.stepglm_4<-glm(as.factor(pa) ~ 
              north_south_asp
              + east_west_asp 
              +GDD_corngrowing_dds_2005
              +PRISM_tmean_30yr_normal_800mM2_annual_asc
              +springs_cd
              +wetlands_cd
              +wtrshd_size
              ,family = binomial(link = "logit"), data=train)
All_Results[4,8]<-threshold(evaluate(pres_test, abs_test, FR.stepglm_4))[2] #this is also done within evaluate
All_Results[4,2]<-evaluate(pres_test, abs_test, FR.stepglm_4)@auc

FR.glm_4<-glm(as.factor(pa) ~.,family = binomial(link = "logit"), data=train)
All_Results[4,9]<-threshold(evaluate(pres_test, abs_test, FR.glm_4))[2] #this is also done within evaluate
All_Results[4,3]<-evaluate(pres_test, abs_test, FR.glm_4)@auc

#GAM
#not sure how to properly set this up
FR.gam_4<-gam(as.factor(pa) ~ s(east_west_asp)+
                s(north_south_asp)+
                s(GDD_corngrowing_dds_2005)+
                s(NPP_mean_00_15)+
                s(PRISM_tmean_30yr_normal_800mM2_annual_asc)+
                s(slope)+
                s(springs_cd)+
                s(streams_cd)+
                s(wetlands_cd)+
                s(wtrshd_size)
              ,family = binomial(link = "logit"), data=train, select=T)
All_Results[4,10]<-threshold(evaluate(pres_test, abs_test, FR.gam_4))[2]
All_Results[4,4]<-evaluate(pres_test, abs_test, FR.gam_4)@auc

maxent_train<-data.frame(rbind(pres_train,abs_train))
me_pa<-maxent_train[,1]
maxent_train<-maxent_train[,-1]
jar <- paste(system.file(package="dismo"), "/java/maxent.jar", sep='')
if(file.exists(jar)){
  FR.maxent_4<-maxent(x=maxent_train,p=me_pa,path="./FR.MaxEnt/4")
}
#evaluate(pres_test, abs_test, FR.maxent)
All_Results[4,5]<-evaluate(pres_test, abs_test, FR.maxent_4)@auc
All_Results[4,11]<-threshold(evaluate(pres_test, abs_test, FR.maxent_4))[2]

#Random Forest
model <- pa ~ .
FR.randfor_4 <- randomForest(model, data=na.omit(train), importance=T)

#evaluate(pres_test, abs_test, FR.randfor)
All_Results[4,6]<-evaluate(pres_test, abs_test, FR.randfor_4)@auc
All_Results[4,12]<-threshold(evaluate(pres_test, abs_test, FR.randfor_4))[2]

#Hyper Volumes
## Setup xgboost data structures
## This first line converts categorical variables to proper dummy variables
train.x <- sparse.model.matrix(pa~.-1, data = train)
train.y = train[,"pa"]
test.x <- sparse.model.matrix(pa~.-1, data = test)
test.y = test[,"pa"]
## Run xgboost
## max.depth: size of trees
## eta: gradient step
## nthread: cpu threads used
## nrounds: number of iterations (increase this if eta is decreased)
## objective: type of training algorithm
FR.bst_4 <- xgboost(data = train.x, label = train.y, max_depth = 4,
                    eta = 0.25, nthread = 2, nrounds=500,objective = "binary:logistic")
fit.pred <- predict(FR.bst_4, newdata=test.x)
fit.ev <- evaluate(p=fit.pred[test.y==1], a=fit.pred[test.y==0])
All_Results[4,7] <- fit.ev@auc
All_Results[4,13] <- threshold(fit.ev)$spec_sens
```

# 6 Results

The table below shows the AUC scores of each model created above. GLM has the lowest AUC, while GAM and MaxEnt are similiar, and Random Forest and XGboost are essentially the same.

```
All_Results[,2:7]
```

```
##   stepGLM_AUC   GLM_AUC   GAM_AUC    ME_AUC    RF_AUC    XG_AUC
## 1   0.7792089 0.7803788 0.8775688 0.8908760 0.9374808 0.9240019
## 2   0.7787384 0.7793973 0.8672922 0.8735792 0.9235437 0.9171254
## 3   0.7781017 0.7787519 0.8615302 0.8841005 0.9277419 0.9261060
## 4   0.7709928 0.7687713 0.8534635 0.8802241 0.9190153 0.9135286
```

```
#summary(All_Results)

boxplot(All_Results$stepGLM_AUC,All_Results$GLM_AUC, All_Results$GAM_AUC, All_Results$ME_AUC, All_Results$RF_AUC, notch=F, names=c(names(All_Results[2:6])), main="Model AUC Scores")
```

This allows for use to see the range of AUC variation (which should be small between folds) between model approaches to determine which model has the greatest predictive power.

To determine if increases in AUC are significant we use an ANOVA and Tukey HSD.

```
#dataframe creation for test
anov<-data.frame(auc=unlist(All_Results[,2:7]))
anov$model<-rownames(anov);rownames(anov)<-c()
anov$model<- c(rep("stepGLM",4),rep("GLM",4),rep("GAM",4),rep("MaxEnt",4),rep("RF",4),rep("XG",4))
#ANOVA
summary(aov(auc~model, data=anov))
```

```
##             Df  Sum Sq  Mean Sq F value Pr(>F)    
## model        5 0.08978 0.017955   364.1 <2e-16 ***
## Residuals   18 0.00089 0.000049                   
## ---
## Signif. codes:  0 '***' 0.001 '**' 0.01 '*' 0.05 '.' 0.1 ' ' 1
```

```
#Tukey HSD
TukeyHSD(aov(auc~model, data=anov))
```

```
##   Tukey multiple comparisons of means
##     95% family-wise confidence level
## 
## Fit: aov(formula = auc ~ model, data = anov)
## 
## $model
##                         diff          lwr         upr     p adj
## GLM-GAM        -8.813888e-02 -0.103919554 -0.07235821 0.0000000
## MaxEnt-GAM      1.723128e-02  0.001450612  0.03301195 0.0277809
## RF-GAM          6.198172e-02  0.046201053  0.07776239 0.0000000
## stepGLM-GAM    -8.820325e-02 -0.103983921 -0.07242258 0.0000000
## XG-GAM          5.522679e-02  0.039446122  0.07100746 0.0000000
## MaxEnt-GLM      1.053702e-01  0.089589494  0.12115084 0.0000000
## RF-GLM          1.501206e-01  0.134339935  0.16590128 0.0000000
## stepGLM-GLM    -6.436702e-05 -0.015845038  0.01571630 1.0000000
## XG-GLM          1.433657e-01  0.127585004  0.15914635 0.0000000
## RF-MaxEnt       4.475044e-02  0.028969770  0.06053111 0.0000006
## stepGLM-MaxEnt -1.054345e-01 -0.121215203 -0.08965386 0.0000000
## XG-MaxEnt       3.799551e-02  0.022214839  0.05377618 0.0000060
## stepGLM-RF     -1.501850e-01 -0.165965644 -0.13440430 0.0000000
## XG-RF          -6.754931e-03 -0.022535602  0.00902574 0.7487798
## XG-stepGLM      1.434300e-01  0.127649372  0.15921071 0.0000000
```

```
plot(TukeyHSD(aov(auc~model, data=anov)),las=1,cex.axis=.5)
```

The ANOVA indicates that there are differences in AUC between modeling approaches and the Tukey HSD shows where these differences are found. Looking at the p-values, we see differences in AUC between every modeling approach except between XG Boost and Random Forest, and the stepGLM and GLM. The results indicate that Random Forest modeling approach produces the most powerful predictive models (see main article for why MaxEnt is the appropriate tool even though RF results in a higher AUC).

## 6.1 Variable Contribution

Variable contribution is measured differently between modeling approaches. As such, they are not directly comparable, but this is okay. We are more interested in the rank order of the contributing variables to see if different modeling approaches found similiar contributions of predictor variables.

```
variable_cont<-data.frame(Variable=c("Mean Temp", "NPP", "Springs_CD", "Wetlands_CD", "GDD", "Watershed_Size", "Slope", "Northish", "Streams_CD", "Eastish"), StepGLM=NA, GLM=NA, GAM=NA, MaxEnt=c(1:10), RF=NA)
```

### 6.1.1 GLM

We assess variable contributions in GLM using beta coefficients. The beta coefficients are the change in logit unit space and can be interpretted as the slope of the line with more significant variables having steeper slopes. Because our variables are in different units of measurement, some ranging from 0 to 1,000 and others ranging from -1 to 1, we need to standardize the beta coefficients to make them comparable within the GLM. To do this, we need to re-run the model and scale our variables using `scale`. This will normalize the response variable’s predictor values. Then we need to convert the beta coefficients from logit space to

GLM variable contributions vary slightly between models, but hold the same relative rank order with GDD, Watershed size, cost distance to springs, cost distance to wetlands, and mean temperature. All variables are significant.

```
#kfold 1 data

train <- sdmdata[train_group != All_Results[1,1], ] #model training gropu, not equal to i
pres_train<-train[train$pa==1,] #all the presence points in the training group
abs_train<-train[train$pa==0,] # all the absence poings in the training group
test <- sdmdata[train_group == All_Results[1,1], ] #model test gropu, equal to i
pres_test<-test[test$pa==1,] #all presence in test data
abs_test<-test[test$pa==0,] #all absence in test data

glm_varbs_1<-glm(as.factor(pa) ~scale(north_south_asp)+
                   scale(east_west_asp)+
                scale(GDD_corngrowing_dds_2005)+
                scale(NPP_mean_00_15)+
                scale(PRISM_tmean_30yr_normal_800mM2_annual_asc)+
                scale(slope)+
                scale(springs_cd)+
                scale(streams_cd)+
                scale(wetlands_cd)+
                scale(wtrshd_size),family = binomial(link = "logit"), data=train)
  exp(glm_varbs_1$coefficients)/1+exp(glm_varbs_1$coefficients)
```

```
##                                      (Intercept) 
##                                        0.1910529 
##                           scale(north_south_asp) 
##                                        1.6550310 
##                             scale(east_west_asp) 
##                                        2.1632503 
##                  scale(GDD_corngrowing_dds_2005) 
##                                        3.6229972 
##                            scale(NPP_mean_00_15) 
##                                        2.0419926 
## scale(PRISM_tmean_30yr_normal_800mM2_annual_asc) 
##                                        1.7115152 
##                                     scale(slope) 
##                                        1.9718689 
##                                scale(springs_cd) 
##                                        1.1166984 
##                                scale(streams_cd) 
##                                        2.0827722 
##                               scale(wetlands_cd) 
##                                        1.1214954 
##                               scale(wtrshd_size) 
##                                        1.3976905
```

```
barplot(exp(glm_varbs_1$coefficients)/1+exp(glm_varbs_1$coefficients),las=2,cex.names=.7)
```

```
#kfold2

train <- sdmdata[train_group != All_Results[2,1], ] #model training gropu, not equal to i
pres_train<-train[train$pa==1,] #all the presence points in the training group
abs_train<-train[train$pa==0,] # all the absence poings in the training group
test <- sdmdata[train_group == All_Results[2,1], ] #model test gropu, equal to i
pres_test<-test[test$pa==1,] #all presence in test data
abs_test<-test[test$pa==0,] #all absence in test data

glm_varbs_2<-glm(as.factor(pa) ~scale(north_south_asp)+
                   scale(east_west_asp)+
                scale(GDD_corngrowing_dds_2005)+
                scale(NPP_mean_00_15)+
                scale(PRISM_tmean_30yr_normal_800mM2_annual_asc)+
                scale(slope)+
                scale(springs_cd)+
                scale(streams_cd)+
                scale(wetlands_cd)+
                scale(wtrshd_size),family = binomial(link = "logit"), data=train)
  exp(glm_varbs_2$coefficients)/1+exp(glm_varbs_2$coefficients)
```

```
##                                      (Intercept) 
##                                        0.1980286 
##                           scale(north_south_asp) 
##                                        1.6480352 
##                             scale(east_west_asp) 
##                                        2.2104038 
##                  scale(GDD_corngrowing_dds_2005) 
##                                        3.5141036 
##                            scale(NPP_mean_00_15) 
##                                        2.0157891 
## scale(PRISM_tmean_30yr_normal_800mM2_annual_asc) 
##                                        1.7602390 
##                                     scale(slope) 
##                                        1.9093291 
##                                scale(springs_cd) 
##                                        1.1378404 
##                                scale(streams_cd) 
##                                        2.0960403 
##                               scale(wetlands_cd) 
##                                        1.0756916 
##                               scale(wtrshd_size) 
##                                        1.3847848
```

```
barplot(exp(glm_varbs_2$coefficients)/1+exp(glm_varbs_2$coefficients),las=2,cex.names=.7)
```

```
#kfold3

train <- sdmdata[train_group != All_Results[3,1], ] #model training gropu, not equal to i
pres_train<-train[train$pa==1,] #all the presence points in the training group
abs_train<-train[train$pa==0,] # all the absence poings in the training group
test <- sdmdata[train_group == All_Results[3,1], ] #model test gropu, equal to i
pres_test<-test[test$pa==1,] #all presence in test data
abs_test<-test[test$pa==0,] #all absence in test data

glm_varbs_3<-glm(as.factor(pa) ~scale(north_south_asp)+
                   scale(east_west_asp)+
                scale(GDD_corngrowing_dds_2005)+
                scale(NPP_mean_00_15)+
                scale(PRISM_tmean_30yr_normal_800mM2_annual_asc)+
                scale(slope)+
                scale(springs_cd)+
                scale(streams_cd)+
                scale(wetlands_cd)+
                scale(wtrshd_size),family = binomial(link = "logit"), data=train)
  exp(glm_varbs_3$coefficients)/1+exp(glm_varbs_3$coefficients)
```

```
##                                      (Intercept) 
##                                        0.1915042 
##                           scale(north_south_asp) 
##                                        1.6694073 
##                             scale(east_west_asp) 
##                                        2.2355983 
##                  scale(GDD_corngrowing_dds_2005) 
##                                        3.4602877 
##                            scale(NPP_mean_00_15) 
##                                        2.0394321 
## scale(PRISM_tmean_30yr_normal_800mM2_annual_asc) 
##                                        1.7662161 
##                                     scale(slope) 
##                                        1.9436866 
##                                scale(springs_cd) 
##                                        1.1633520 
##                                scale(streams_cd) 
##                                        2.0760551 
##                               scale(wetlands_cd) 
##                                        1.0556545 
##                               scale(wtrshd_size) 
##                                        1.3413964
```

```
  barplot(exp(glm_varbs_3$coefficients)/1+exp(glm_varbs_3$coefficients),las=2,cex.names=.7)
```

```
#kfold4

train <- sdmdata[train_group != All_Results[4,1], ] #model training gropu, not equal to i
pres_train<-train[train$pa==1,] #all the presence points in the training group
abs_train<-train[train$pa==0,] # all the absence poings in the training group
test <- sdmdata[train_group == All_Results[4,1], ] #model test gropu, equal to i
pres_test<-test[test$pa==1,] #all presence in test data
abs_test<-test[test$pa==0,] #all absence in test data

glm_varbs_4<-glm(as.factor(pa) ~scale(north_south_asp)+
                   scale(east_west_asp)+
                scale(GDD_corngrowing_dds_2005)+
                scale(NPP_mean_00_15)+
                scale(PRISM_tmean_30yr_normal_800mM2_annual_asc)+
                scale(slope)+
                scale(springs_cd)+
                scale(streams_cd)+
                scale(wetlands_cd)+
                scale(wtrshd_size),family = binomial(link = "logit"), data=train)
  exp(glm_varbs_4$coefficients)/1+exp(glm_varbs_4$coefficients)
```

```
##                                      (Intercept) 
##                                        0.2030648 
##                           scale(north_south_asp) 
##                                        1.5976429 
##                             scale(east_west_asp) 
##                                        2.2323644 
##                  scale(GDD_corngrowing_dds_2005) 
##                                        3.5432557 
##                            scale(NPP_mean_00_15) 
##                                        2.0001833 
## scale(PRISM_tmean_30yr_normal_800mM2_annual_asc) 
##                                        1.7009795 
##                                     scale(slope) 
##                                        1.8698916 
##                                scale(springs_cd) 
##                                        1.1126530 
##                                scale(streams_cd) 
##                                        2.1467995 
##                               scale(wetlands_cd) 
##                                        1.1375871 
##                               scale(wtrshd_size) 
##                                        1.3565915
```

```
  barplot(exp(glm_varbs_4$coefficients)/1+exp(glm_varbs_4$coefficients),las=2,cex.names=.7)
```

### 6.1.2 GAM

We measure variable contribution for the GAM using the chi-squared statistic.

```
barplot(summary(FR.gam_1)$chi.sq,las=2,cex.names=.7)
```

```
barplot(summary(FR.gam_2)$chi.sq,las=2,cex.names=.7)
```

```
barplot(summary(FR.gam_3)$chi.sq,las=2,cex.names=.7)
```

```
barplot(summary(FR.gam_4)$chi.sq,las=2,cex.names=.7)
```

### 6.1.3 MaxEnt

MaxEnt is slightly different and the models are saved within the model as an HTML if you specify output. See folder `FR.MaxEnt`.

```
plot(FR.maxent_1)
```

```
response(FR.maxent_1)
```

```
plot(FR.maxent_2)
```

```
plot(FR.maxent_3)
```

```
plot(FR.maxent_4)
```

### 6.1.4 Random Forest

Variable contribution in random forests are assess using the percent increase in mean squared error (%incMSE). %incMSE for each variable is determined by leaving out the variable from the model and measuring the effect on MSE. More important variables will have higher %incMSE.

```
#FR.randfor_1$importance
barplot(FR.randfor_1$importance[,1],las=2,cex.names=.7,ylab="%IncMSE")
```

```
#FR.randfor_2$importance
barplot(FR.randfor_2$importance[,1],las=2,cex.names=.7,ylab="%IncMSE")
```

```
#FR.randfor_3$importance
barplot(FR.randfor_3$importance[,1],las=2,cex.names=.7,ylab="%IncMSE")
```

```
#FR.randfor_4$importance
barplot(FR.randfor_4$importance[,1],las=2,cex.names=.7,ylab="%IncMSE")
```

Looking at the results, GAM, and MaxEnt all identified *Mean annual temperature* and *NPP* as the prime contributors, Random Forest identified *Mean annual temperature*, *GDD*, and *NPP*, and GLM identified *GDD* and *Eastish* as prime contributors.

# 7 Predictive Maps

Using our models, we will now create continous probability distribution maps. These are the predictive layers. They are created using the predictor variables and the model results. Because we created 4 models for each modeling approach, we will generate a predictive layer for each model in each approach and then stack them and take the average probabilities for our final model.

We evaluate the prediction maps using all the presence and absence points to calculate the AUC. Because the predcition maps are created using all models of each approach, and thus all data, in this application the AUC cannot be interpretted as a measure of model performance, but rather a goodness-of-fit for the existing data.

Creating the predictive rasters can be time consuming on large predictor variable sets.

## 7.1 GLM Predictive Map

For example, below we have the four GLM models. We create a predictive layer using each model type. We then stack the predictive layers and calculate the average to create a new predictive surface. We then calculate a final AUC using all presence and absence points. This is done for each model approach.

```
pred_glm_1<-raster::predict(model=FR.glm_1, object=predictors,type="response")
pred_glm_2<-raster::predict(model=FR.glm_2, object=predictors,type="response")
pred_glm_3<-raster::predict(model=FR.glm_3, object=predictors,type="response")
pred_glm_4<-raster::predict(model=FR.glm_4, object=predictors,type="response")

pred_glms<-stack(pred_glm_1, pred_glm_2, pred_glm_3, pred_glm_4)
GLM_Pred<-mean(pred_glms)
GLM_SD
```

```
plot(GLM_Pred)
plot(GSENM_B, add=T)
```

```
p_glm<-extract(GLM_Pred,pres)
a_glm<-extract(GLM_Pred,abs)
```

```
evaluate(p=p_glm,a=a_glm)
```

```
## class          : ModelEvaluation 
## n presences    : 1619 
## n absences     : 10195 
## AUC            : 0.7780643 
## cor            : 0.3755887 
## max TPR+TNR at : 0.1390384
```

```
par(pty="s")
plot(evaluate(p=p_glm,a=a_glm),'ROC',type="l")
```

```
#plot(evaluate(p=p_glm,a=a_glm),'TPR')
#abline(v=0.17)
#plot(evaluate(p=p_glm,a=a_glm),'TNR')
#abline(v=0.17)
```

By default `predict` produces results in log-odds scale than in probability. To specify that we want a the results in a probability scale, we only need to specify `type="response"` as a parameter. Note that we do not need to do this for MaxEnt or Random Forest.

## 7.2 GAM Predictive Map

```
pred_gam_1<-raster::predict(model=FR.gam_1, object=predictors,type="response")
pred_gam_2<-raster::predict(model=FR.gam_2, object=predictors,type="response")
pred_gam_3<-raster::predict(model=FR.gam_3, object=predictors,type="response")
pred_gam_4<-raster::predict(model=FR.gam_4, object=predictors,type="response")

pred_gams<-stack(pred_gam_1, pred_gam_2, pred_gam_3, pred_gam_4)
GAM_Pred<-mean(pred_gams)
```

```
plot(GAM_Pred)
plot(GSENM_B, add=T)
```

```
p_gam<-extract(GAM_Pred,pres)
a_gam<-extract(GAM_Pred,abs)
```

```
evaluate(p=p_gam,a=a_gam)
```

```
## class          : ModelEvaluation 
## n presences    : 1619 
## n absences     : 10195 
## AUC            : 0.8735646 
## cor            : 0.581551 
## max TPR+TNR at : 0.1291189
```

```
par(pty="s")
plot(evaluate(p=p_gam,a=a_gam),'ROC',type='l')
```

```
#plot(evaluate(p=p_gam,a=a_gam),'TPR')
```

## 7.3 MaxEnt Predictive Map

```
pred_maxent_1<-predict(FR.maxent_1, predictors)
pred_maxent_2<-predict(FR.maxent_2, predictors)
pred_maxent_3<-predict(FR.maxent_3, predictors)
pred_maxent_4<-predict(FR.maxent_4, predictors)

pred_maxent<-stack(pred_maxent_1, pred_maxent_2, pred_maxent_3, pred_maxent_4)
MaxEnt_Pred<-mean(pred_maxent)
```

```
plot(MaxEnt_Pred)
plot(GSENM_B, add=T)
```

```
p_me<-extract(MaxEnt_Pred,pres)
a_me<-extract(MaxEnt_Pred,abs)
```

```
evaluate(p=p_me,a=a_me)
```

```
## class          : ModelEvaluation 
## n presences    : 1619 
## n absences     : 10195 
## AUC            : 0.8968426 
## cor            : 0.5944039 
## max TPR+TNR at : 0.4109781
```

```
par(pty="s")
plot(evaluate(p=p_me,a=a_me),'ROC',type='l')
```

```
#plot(evaluate(p=p_me,a=a_me),'TPR')
#abline(v=0.44)
#plot(evaluate(p=p_me,a=a_me),'TNR')
#abline(v=0.44)
```

## 7.4 Random Forest Predictive Map

```
pred_rf_1<-raster::predict(model=FR.randfor_1, object=predictors)
pred_rf_2<-raster::predict(model=FR.randfor_2, object=predictors)
pred_rf_3<-raster::predict(model=FR.randfor_3, object=predictors)
pred_rf_4<-raster::predict(model=FR.randfor_4, object=predictors)

pred_rfs<-stack(pred_rf_1, pred_rf_2, pred_rf_3, pred_rf_4)
RF_Pred<-mean(pred_rfs)
```

```
plot(RF_Pred)
```

```
## Warning in showSRID(uprojargs, format = "PROJ", multiline = "NO"): Discarded datum Unknown based on GRS80 ellipsoid in CRS definition,
##  but +towgs84= values preserved

## Warning in showSRID(uprojargs, format = "PROJ", multiline = "NO"): Discarded datum Unknown based on GRS80 ellipsoid in CRS definition,
##  but +towgs84= values preserved

## Warning in showSRID(uprojargs, format = "PROJ", multiline = "NO"): Discarded datum Unknown based on GRS80 ellipsoid in CRS definition,
##  but +towgs84= values preserved

## Warning in showSRID(uprojargs, format = "PROJ", multiline = "NO"): Discarded datum Unknown based on GRS80 ellipsoid in CRS definition,
##  but +towgs84= values preserved
```

```
plot(GSENM_B, add=T)
```

```
p_rf<-extract(RF_Pred,pres)
a_rf<-extract(RF_Pred,abs)
```

```
evaluate(p=p_rf,a=a_rf)
```

```
## class          : ModelEvaluation 
## n presences    : 1619 
## n absences     : 10195 
## AUC            : 0.9990328 
## cor            : 0.9312344 
## max TPR+TNR at : 0.3732665
```

```
par(pty="s")
plot(evaluate(p=p_rf,a=a_rf),'ROC',type='l')
```

```
#plot(evaluate(p=p_rf,a=a_rf),'TPR')
#abline(v=0.38)
#plot(evaluate(p=p_rf,a=a_rf),'TNR')
#abline(v=0.38)
```

As we can see, there are differences in the predictions made by each modeling approach. In general, GLM produce predictive maps that assign much more potential for Formative Period land use than GAM, MaxEnt, and Random Forest. GAM and Maxent are similiar in thier predictions but with MaxEnt having a better model result and better goodness-of-fit. Random Forest is by far the most conservative of the modeling approaches and assigns high probabilities to a relatively small area.

# 8 Binary Maps

Often, archaeologists convert the continous probability distributions produced by models into binary. 0 represents the predicted absence of a site and 1 represents the predicted presence of a site. The danger here is that it converts continous probabilities to certainties. It is our belief that the continous probability maps are more useful when assessing the likelihood of a site, but at the cost of ease of interpretation.

To convert the continous probability distributions to binary, we first need to determine a threshold. A threshold is the probability at which all values below are assigned a 0 and all values above are assigned a 1. The justificaiton for threshold values seems aritrary in much of archaeology. The importance of thresholds has to do with a models sensitivity and specificity. These are the True Positive Rate and the True Negative Rate. By understanding the relationship of these rates and how threshold values alter the two is important. Here, we advocate for an optimal threshold. The optimal threshold is the probability at which the TPR and TNR are maximized. The optimal threshold is calculated in the previous section during predictive map evaluation.

Binary maps are common in archaeology (see our discussion in the paper). Unfortunately they result in a loss of information by converting the continous probability distribution into 0 or 1. The conversion to 0 or 1 is almost arbitrary and is dependent on a threshold value. As noted above, we calculated the optimal threshold (threshold of max TPR+TNR) values for each model run and now we will use the optimal threshold determined from the final evaluations of the predictive maps above to create binary maps.

Below we convert the Random Forest Predictive map to binary. We create a function that will reclasify values less than or equal to the optimal threshold (0.38 as determined above) as 0, for absence, and values above the optimal threshold as 1, for presence. In addition, we provide some “standard” archaeological theshold-dependent measures.

## 8.1 GLM Binary Map

```
binary_reclassify <- function(x) {
  ifelse(x <=  0.139, 0,
  ifelse(x >  0.139, 1, NA)) 
}

GLM_binary <- calc(GLM_Pred, fun=binary_reclassify)
```

```
plot(GLM_binary)
plot(GSENM_B, add=T)
```

```
#table where we will store all the stats
stats_table<-data.frame(Model=c("GLM","GAM","ME","RF"),
                        TSS=NA,
                        Kappa=NA,
                        "p(S|M)/p(S|'M)"=NA,
                        "p(M|S)"=NA,
                        "p(S)"=NA,
                        "p(M)"=NA,
                        "p(M)/p(S)"=NA,
                        "p(M|S)-p(M)"=NA,
                        "p(S|M)/p(S)"=NA)
stats_table[1,2]<-(evaluate(p=p_glm,a=a_glm,tr=0.139)@TPR + evaluate(p=p_glm,a=a_glm,tr=0.139)@TNR) -1
#Kappa
stats_table[1,3]<-evaluate(p=p_glm,a=a_glm,tr=0.139)@kappa

###
#Archaeology Stats:
#ArchStats<-data.frame(GLM=rep(NA,1619),GAM=NA,MaxEnt=NA, RF=NA)
#ArchStats[,1]<-extract(GLM_binary,y=pres,method="simple")
#ArchStats[,2]<-extract(GAM_binary,y=pres,method="simple")
#ArchStats[,3]<-extract(ME_binary,y=pres,method="simple")
#ArchStats[,4]<-extract(RF_binary,y=pres,method="simple")

#Percent Correct Statistic/Model Accuracy: (p(M|S))
stats_table[1,5]<-sum(ArchStats[,1])/nrow(ArchStats)
#Probability of Occurence - All models must do better than this else it is worthless. (p(S))
stats_table[1,6]<-nrow(ArchStats)/ncell(GLM_binary)
#Probability of absence (p(S'))
1-nrow(ArchStats)/ncell(GLM_binary)
#Base rate that model will indicate a site (p(M))
stats_table[1,7]<-cellStats(GLM_binary,sum)/ncell(GLM_binary)
#Base rate that a model will indicate the absence of a site - which can be described as Model Precision (higher is better) (p(M'))
1-(cellStats(GLM_binary,sum)/ncell(GLM_binary))
#Index of Model Fit (p(M)/p(S)) (want small numbers)
stats_table[1,8]<-(cellStats(GLM_binary,sum)/ncell(GLM_binary))/(nrow(ArchStats)/ncell(GLM_binary))

#Improvement over Chance (p(M|S)-p(M)
stats_table[1,9]<-(sum(ArchStats[,1])/nrow(ArchStats))-(cellStats(GLM_binary,sum)/ncell(GLM_binary))
#Probability of Arch site present when model specifies (p(S|M))
(sum(ArchStats[,1]))/(cellStats(GLM_binary,sum))
#Probabiliyt of Arch site presence when model specifies absence.  (p(S|M'))
(sum(ArchStats[,1]))/(ncell(GLM_binary)-cellStats(GLM_binary,sum))
#Model Improvement Ratio (p(S|M)/p(S))
stats_table[1,10]<-((sum(ArchStats[,1]))/(cellStats(GLM_binary,sum)))/(nrow(ArchStats)/ncell(GLM_binary))
#Model Improvement Ratio with absences (p(S)/p(S|M'))
(nrow(ArchStats)/ncell(GLM_binary))/(sum(ArchStats[,1]))/(ncell(GLM_binary)-cellStats(GLM_binary,sum))
#Model IMprovement Ratio: p(S|M)/p(S|M')
stats_table[1,4]<-((sum(ArchStats[,1]))/(cellStats(GLM_binary,sum)))/((sum(ArchStats[,1]))/(ncell(GLM_binary)-cellStats(GLM_binary,sum)))
```

## 8.2 GAM Binary Map

```
binary_reclassify <- function(x) {
  ifelse(x <=  0.129, 0,
  ifelse(x >  0.129, 1, NA)) 
}

GAM_binary <- calc(GAM_Pred, fun=binary_reclassify)
```

```
plot(GAM_binary)
plot(GSENM_B, add=T)
```

```
#TSS (TPR + TNR - 1)
stats_table[2,2]<-(evaluate(p=p_gam,a=a_gam,tr=0.129)@TPR + evaluate(p=p_gam,a=a_gam,tr=0.129)@TNR) -1
#Kappa
stats_table[2,3]<-evaluate(p=p_gam,a=a_gam,tr=0.129)@kappa

###
#Archaeology Stats:
#Percent Correct Statistic/Model Accuracy: (p(M|S))
stats_table[2,5]<-sum(ArchStats[,2])/nrow(ArchStats)
#Probability of Occurence - All models must do better than this else it is worthless. (p(S))
stats_table[2,6]<-nrow(ArchStats)/ncell(GAM_binary)
#Probability of absence (p(S'))
1-nrow(ArchStats)/ncell(GAM_binary)
#Base rate that model will indicate a site (p(M))
stats_table[2,7]<-cellStats(GAM_binary,sum)/ncell(GAM_binary)
#Base rate that a model will indicate the absence of a site - which can be described as Model Precision (higher is better) (p(M'))
1-(cellStats(GAM_binary,sum)/ncell(GAM_binary))
#Index of Model Fit (p(M)/p(S) (want small numbers)
stats_table[2,8]<-(cellStats(GAM_binary,sum)/ncell(GAM_binary))/(nrow(ArchStats)/ncell(GAM_binary))

#Improvement over Chance (p(M|S)-p(M)
stats_table[2,9]<-(sum(ArchStats[,2])/nrow(ArchStats))-(cellStats(GAM_binary,sum)/ncell(GAM_binary))
#Probability of Arch site present when model specifies (p(S|M))
(sum(ArchStats[,2]))/(cellStats(GAM_binary,sum))
#Probabiliyt of Arch site presence when model specifies absence.  (p(S|M'))
(sum(ArchStats[,2]))/(ncell(GAM_binary)-cellStats(GAM_binary,sum))
#Model Improvement Ratio (p(S|M)/p(S))
stats_table[2,10]<-((sum(ArchStats[,2]))/(cellStats(GAM_binary,sum)))/(nrow(ArchStats)/ncell(GAM_binary))
#Model Improvement Ratio with absences (p(S)/p(S|M'))
(nrow(ArchStats)/ncell(GAM_binary))/(sum(ArchStats[,2]))/(ncell(GAM_binary)-cellStats(GAM_binary,sum))
#Model IMprovement Ratio: p(S|M)/p(S|M')
stats_table[2,4]<-((sum(ArchStats[,2]))/(cellStats(GAM_binary,sum)))/((sum(ArchStats[,2]))/(ncell(GAM_binary)-cellStats(GAM_binary,sum)))
```

## 8.3 Maxent Binary Map

```
binary_reclassify <- function(x) {
  ifelse(x <=  0.411, 0,
  ifelse(x >  0.411, 1, NA)) 
}

ME_binary <- calc(MaxEnt_Pred, fun=binary_reclassify)
```

```
plot(ME_binary)
plot(GSENM_B, add=T)
```

```
#TSS (TPR + TNR - 1)
stats_table[3,2]<-(evaluate(p=p_me,a=a_me,tr=0.411)@TPR + evaluate(p=p_me,a=a_me,tr=0.411)@TNR) -1
#Kappa
stats_table[3,3]<-evaluate(p=p_me,a=a_me,tr=0.411)@kappa

###
#Archaeology Stats:
#Percent Correct Statistic/Model Accuracy: (p(M|S))
stats_table[3,5]<-sum(ArchStats[,3])/nrow(ArchStats)
#Probability of Occurence - All models must do better than this else it is worthless. (p(S))
stats_table[3,6]<-nrow(ArchStats)/ncell(ME_binary)
#Probability of absence (p(S'))
1-nrow(ArchStats)/ncell(ME_binary)
#Base rate that model will indicate a site (p(M))
stats_table[3,7]<-cellStats(ME_binary,sum)/ncell(ME_binary)
#Base rate that a model will indicate the absence of a site - which can be described as Model Precision (higher is better) (p(M'))
1-(cellStats(ME_binary,sum)/ncell(ME_binary))
#Index of Model Fit (p(M)/p(S) (want small numbers)
stats_table[3,8]<-(cellStats(ME_binary,sum)/ncell(ME_binary))/(nrow(ArchStats)/ncell(ME_binary))

#Improvement over Chance (p(M|S)-p(M)
stats_table[3,9]<-(sum(ArchStats[,3])/nrow(ArchStats))-(cellStats(ME_binary,sum)/ncell(ME_binary))
#Probability of Arch site present when model specifies (p(S|M))
(sum(ArchStats[,3]))/(cellStats(ME_binary,sum))
#Probability of Arch site presence when model specifies absence.  (p(S|M'))
(sum(ArchStats[,3]))/(ncell(ME_binary)-cellStats(ME_binary,sum))
#Model Improvement Ratio (p(S|M)/p(S))
stats_table[3,10]<-((sum(ArchStats[,3]))/(cellStats(ME_binary,sum)))/(nrow(ArchStats)/ncell(ME_binary))
#Model Improvement Ratio with absences (p(S)/p(S|M'))
(nrow(ArchStats)/ncell(ME_binary))/(sum(ArchStats[,3]))/(ncell(ME_binary)-cellStats(ME_binary,sum))
#Model IMprovement Ratio: p(S|M)/p(S|M')
stats_table[3,4]<-((sum(ArchStats[,3]))/(cellStats(ME_binary,sum)))/((sum(ArchStats[,3]))/(ncell(ME_binary)-cellStats(ME_binary,sum)))
```

## 8.4 Random Forest Binary Map

```
binary_reclassify <- function(x) {
  ifelse(x <=  0.373, 0,
  ifelse(x >  0.373, 1, NA)) 
}

RF_binary <- calc(RF_Pred, fun=binary_reclassify)
```

```
plot(RF_binary)
plot(GSENM_B, add=T)
```

```
#Evaluating using TSS (True skill statistic and Cohen's Kappa)
#TSS (TPR + TNR - 1)
stats_table[4,2]<-(evaluate(p=p_rf,a=a_rf,tr=0.373)@TPR + evaluate(p=p_rf,a=a_rf,tr=0.373)@TNR) -1
#Kappa
stats_table[4,3]<-evaluate(p=p_rf,a=a_rf,tr=0.373)@kappa

###
#Archaeology Stats:
#Percent Correct Statistic/Model Accuracy: (p(M|S))
stats_table[4,5]<-sum(ArchStats[,4])/nrow(ArchStats)
#Probability of Occurence - All models must do better than this else it is worthless. (p(S))
stats_table[4,6]<-nrow(ArchStats)/ncell(RF_binary)
#Probability of absence (p(S'))
1-nrow(ArchStats)/ncell(RF_binary)
#Base rate that model will indicate a site (p(M))
stats_table[4,7]<-cellStats(RF_binary,sum)/ncell(RF_binary)
#Base rate that a model will indicate the absence of a site - which can be described as Model Precision (higher is better) (p(M'))
1-(cellStats(RF_binary,sum)/ncell(RF_binary))
#Index of Model Fit (p(M)/p(S) (want small numbers)
stats_table[4,8]<-(cellStats(RF_binary,sum)/ncell(RF_binary))/(nrow(ArchStats)/ncell(RF_binary))

#Improvement over Chance (p(M|S)-p(M)
stats_table[4,9]<-(sum(ArchStats[,4])/nrow(ArchStats))-(cellStats(RF_binary,sum)/ncell(RF_binary))
#Probability of Arch site present when model specifies (p(S|M))
(sum(ArchStats[,4]))/(cellStats(RF_binary,sum))
#Probabiliyt of Arch site presence when model specifies absence.  (p(S|M'))
(sum(ArchStats[,4]))/(ncell(RF_binary)-cellStats(RF_binary,sum))
#Model Improvement Ratio (p(S|M)/p(S))
stats_table[4,10]<-((sum(ArchStats[,4]))/(cellStats(RF_binary,sum)))/(nrow(ArchStats)/ncell(RF_binary))
#Model Improvement Ratio with absences (p(S)/p(S|M'))
(nrow(ArchStats)/ncell(RF_binary))/(sum(ArchStats[,4]))/(ncell(RF_binary)-cellStats(RF_binary,sum))
#Model IMprovement Ratio: p(S|M)/p(S|M')
stats_table[4,4]<-((sum(ArchStats[,4]))/(cellStats(RF_binary,sum)))/((sum(ArchStats[,4]))/(ncell(RF_binary)-cellStats(RF_binary,sum)))
```

## 8.5 Evaluations

Below are threshold-dependent statisitics commonly used by ecologists (columns 2-3) and archaeologists (columns 4-10). These statistics are are dependent on a threshold. We select an optimal threshold and reclassify the probability distributions into 0 (absence) and 1 (presence). As you can see in the table below, these statistics support the conclusions made using the threshold-independent measure AUC. RF performs the best, but so well that there is something obviously wrong. The overfitting issue is apparent.

**Column 2:** TSS - True-positive rate + True-negative rate - 1: See Allouche, Tsoar, and Kadmon, 2006.

**Column 3:** Kappa - Measures the improvement over chance. See Allouche, Tsoar, and Kadmon, 2006.

**Column 4:** p(S|M)/p(S|M’) - Model IMprovement Ratio: Indicates how many times more likely a site is in cells predicted as presence versus predicted as absence.

**Column 5:** p(M|S) - Model Accuracy: Probability that a model will correctly indicate a site.

**Column 6:** p(S) - Base rate or chance probability of archaeological site in study region.

**Column 7:** p(M) - Base rate or chance probability that a model will indicate a site.

**Column 8:** p(M)/p(S) - Model Fit: Indicates how many times larger a model mapping is than the total site-class area.

**Column 9:** p(M|S)-p(M) - Model Improvement: Improvement that model offers over chance in specifying known archaeological sites.

**Column 10:** p(S|M)/p(S) - Model improvement Ratio: Indicates how many times more likely a site is in predicted presence locations than the base-rate site probability.

```
stats_table
```

```
##   Model       TSS     Kappa p.S.M..p.S..M.    p.M.S.         p.S.       p.M.
## 1   GLM 0.4360586 0.2472297       1.270141 0.7621989 2.723255e-06 0.44050129
## 2   GAM 0.5809669 0.3914221       5.328952 0.7961705 2.723255e-06 0.15800405
## 3    ME 0.6504760 0.4868224       7.592811 0.8079061 2.723255e-06 0.11637636
## 4    RF 0.9873176 0.9569866      35.110676 0.9993823 2.723255e-06 0.02769264
##   p.M..p.S. p.M.S..p.M. p.S.M..p.S.
## 1 161755.43   0.3216976    1.730299
## 2  58020.29   0.6381664    5.038924
## 3  42734.28   0.6915298    6.942184
## 4  10168.95   0.9716897   36.088372
```

We can see in the maps above the binary predictions produced from each predictive maps probability distribution. GLM produces many predicted presence values because of its relatively low threshold. GAM and MaxEnt are comparable, but MaxEnt produces a more conservative prediction of presence points. Again, this is because of MaxEnt’s higher threshold value. Random Forest produces the most conservative values.

# 9 Known Issues

Below are known issues with the analysis above. Where available, fixes are provided.

## 9.1 Abnormalities in the Predictive Maps

There are abnormalities in the GAM, MaxEnt, and RF maps. These abnormalities manifest as increased probabilities in circle like patterns. These abnormalities are a product of the NPP data. The NPP data are remotely sensed. In areas of highly reflective surfaces (e.g. water, desert, some rock outcrops) NPP values result in a null value of 65355. There are a number of these null values found in the NPP layer. Because NPP is a contributing variable for GAM, MaxEnt, and RF, these values of unusally high NPP result in an artifically increased probability in some areas. These artificially increased probabilities result in the abnormalities present in the predictive maps.

### 9.1.1 Impact

The NPP values result in artifically high probabilities in some areas, which are readily aparent in the predictive map. The abnromalities in the predictive map do not alter the results or conclusions of the paper.

### 9.1.2 Solution

The common solution is to replace the null values in NPP (65355) with NA values and then interpolate the values of those areas. We attempted this but encountered another issue. It appears that some areas surrounding these null value areas have abnormally high NPP. We believe this to be a product of reflective surfaces, but it could also be high NPP around reflective surfaces. Additionally, the higher NPP values could be a result of cattle improvements, such as cattle ponds. Without ground truthing, it is difficult to know what is causing the abnormally high NPP values. Rather than attempt to fix the issue by selecting an arbitrary NPP threshold cutoff, we left the predictive maps as-is, since they do not alter the results or conclusions of the paper.

# 10 Appendices

## 10.1 Predictor Variables

Detailed data on predictor data referenced in Section 2. Below is a table that shows all 55 predictor data. The table shows the type of data, the short title, file title, name in R (used in Section 2), data citation where available, URL to data, and the time frame over which the data was collected.

### 10.1.1 Digital Elevation Model (DEM)

**Description:** AGRC has a statewide coverage of 5 meter Auto-Correlated DEMs in addition to some 2 meter areas. The DEMs were created from the imagery collected during the 2006 NAIP and HRO aerial photography flights. The auto-correlation process is not as rigorous as other methods of elevation modeling such as photogrammetry, lidar mapping, radar mapping, etc, and therefore end-users should be aware that anomalies are expected within the elevation dataset. In comparison to the USGS DEM datasets, the 2 and 5-meter DEMs in some areas provides higher resolution and horizontal accuracy but anomalies are present within the data.

**Categorical Type:** Landscape

**Other Designation:** ‘dem\_5m\_gsenm’

**Included in Final Model:** No

**Units:** Meters

**Original Resolution:** 5 meters x 5 meter

**Dates:** NA

**Source:** Utah AGRC: https://gis.utah.gov/data/elevation-and-terrain/

**Citation:** Utah AGRC. “5 Meter Auto-Correlated Elevation Models.” Utah GIS Portal, 2014. https://gis.utah.gov/data/elevation-terrain-data/5-meter-auto-correlated-elevation-models/.

### 10.1.2 Slope

**Description:** Using the DEM raster, we generate a slope raster using the ‘terrain()’ function in ‘R’.

**Categorical Type:** Landscape

**Other Designation:** ‘slope’

**Included in Final Model:** Yes

**Units:** Degrees of slope

**Original Resolution:** 5 meters x 5 meter

**Dates:** NA

**Source:** Utah AGRC: https://gis.utah.gov/data/elevation-and-terrain/

**Citation:** Utah AGRC. “5 Meter Auto-Correlated Elevation Models.” Utah GIS Portal, 2014. https://gis.utah.gov/data/elevation-terrain-data/5-meter-auto-correlated-elevation-models/.

### 10.1.3 Aspect (East-West)

**Description:** Using the DEM data, we generate aspect which represents the direction in degrees that ground surface slopes. Because direction in degrees is non-linear, we decompose aspect into east-west aspect and north-south aspect (see Section 2.2 for more information).

**Categorical Type:** Landscape

**Other Designation:** ‘east\_west\_asp’

**Included in Final Model:** Yes

**Units:** Degrees (but dimensionless after decomposition)

**Original Resolution:** 5 meter x 5 meter

**Dates:** NA

**Source:** Utah AGRC: https://gis.utah.gov/data/elevation-and-terrain/

**Citation:** Utah AGRC. “5 Meter Auto-Correlated Elevation Models.” Utah GIS Portal, 2014. https://gis.utah.gov/data/elevation-terrain-data/5-meter-auto-correlated-elevation-models/.

### 10.1.4 Aspect (North-South)

**Description:** Using the DEM data, we generate aspect which represents the direction in degrees that ground surface slopes. Because direction in degrees is non-linear, we decompose aspect into east-west aspect and north-south aspect (see Section 2.2 for more information).

**Categorical Type:** Landscape

**Other Designation:** ‘north\_south\_asp’

**Included in Final Model:** Yes

**Units:** Degrees (but dimensionless after decomposition)

**Original Resolution:** 5 meter x 5 meter

**Dates:** NA

**Source:** Utah AGRC: https://gis.utah.gov/data/elevation-and-terrain/

**Citation:** Utah AGRC. “5 Meter Auto-Correlated Elevation Models.” Utah GIS Portal, 2014. https://gis.utah.gov/data/elevation-terrain-data/5-meter-auto-correlated-elevation-models/.

### 10.1.5 Mean Actual and Potential Evapotranspiration Rate (Mean ET and PET)

**Description:** MODIS Evapotranspiration Data Set (MOD16).

**Categorical Type:** Environmental Productivity

**Other Designation:** ‘et\_mean\_00\_13\_1’ and ‘PET\_mean\_00\_13’

**Included in Final Model:** No

**Units:** millimeters/year

**Original Resolution:** 800 meter x 800 meter

**Dates:** 2000-2013

**Source:** NASA MODIS: https://modis.gsfc.nasa.gov/data/dataprod/mod16.php

**Citation:** Numerical Terradynamic Simulation Group. “MODIS Global Evapotranspiration Project (MOD16).” University of Montana, July 29, 2013.

### 10.1.6 Growing-degree days

**Description:** Degree-days (DDs) (which are also referred to as “growing degree-days”, “heat units” or “thermal units”) are the summation of temperature over time. We use a standard 50 degree Farhrenit theshold temperature. We use six different calculation methods including, simple average, growing dds, single triangle, double triangle, signgle sine, double sine and heating and cooling degree-days. Information on each of the methods can be found here http://uspest.org/wea/mapmkrdoc.html. Only the growing dds method for corn is used in the final model.

**Categorical Type:** Environmental Productivity

**Other Designation:** ‘GDD\_corngrowing\_dds\_2005’,‘GDD\_doublesine\_2005’, ‘GDD\_doubletriangle\_2005’, ‘GDD\_simpleaverage\_2005’, ‘GDD\_singlesine\_2005’, ‘GDD\_singletriangle\_2005’, ‘Heating\_dds\_2005’.

**Included in Final Model:** Yes (GDD Corngrowing DDS method)

**Units:** days/year

**Original Resolution:** 100 meter x 100 meter

**Dates:** 30 year average

**Source:** Oregon State: http://uspest.org/cgi-bin/usmapmaker.pl

**Citation:** Coop, Len. “Documentation - US Degree-Day Mapping Calculator,” 2014. http://uspest.org/wea/mapmkrdoc.html.

### 10.1.7 Moisture Index

**Description:** The moisture available to plants. Calculated from actual evapotranspiration / potential evapotranspiration.

**Categorical Type:** Environmental Productivity

**Other Designation:** ‘Moisture\_Index1’

**Included in Final Model:** No

**Units:** millimeters / year

**Original Resolution:** 800 meter x 800 meter

**Dates:** 2000 - 2013

**Source:** ET and PET are derived from NASA MODIS: https://modis.gsfc.nasa.gov/data/dataprod/mod16.php

**Citation:** Ramankutty, Navin, Jonathan A. Foley, John Norman, and Kevin McSweeney. “The Global Distribution of Cultivable Lands: Current Patterns and Sensitivity to Possible Climate Change.” Global Ecology and Biogeography 11, no. 5 (2002): 377-92.

### 10.1.8 Net Primary Productivity (NPP)

**Description:** The difference in total chemical energy produced by plants (gross primary production) and chemical energy invested in plant maintenance and growth (also known as respiration).

**Categorical Type:** Environmental Productivity

**Other Designation:** ‘NPP\_mean\_00\_15’

**Included in Final Model:** Yes

**Units:** Kilograms of Carbon per square meter per year

**Original Resolution:** 1 kilometer x 1 kilometer

**Dates:** 2000 - 2015

**Source:** NASA MODIS: https://modis.gsfc.nasa.gov/data/dataprod/mod17.php

**Citation:** Numerical Terradynamic Simulation Group. “MODIS GPP/NPP Project (MOD17).” University of Montana.

### 10.1.9 PRISM Climate Data

**Description:** PRISM datasets provide estimates of six basic climate elements: precipitation (ppt), minimum temperature (tmin), maximum temperature (tmax), mean dew point (tdmean), minimum vapor pressure deficit (vpdmin), and maximum vapor pressure deficit (vpdmax). Two derived variables, mean temperature (tmean) and vapor pressure (vpr), are sometimes included, depending on the dataset.

**Categorical Type:** Climate

**Other Designation:** ‘PRISM\_ppt\_30yr\_normal\_800mM2\_annual\_asc’, ‘PRISM\_tdmean\_30yr\_normal\_800mM2\_annual\_asc’, ‘PRISM\_tmax\_30yr\_normal\_800mM2\_annual\_asc’,‘PRISM\_tmean\_30yr\_normal\_800mM2\_annual\_asc’, ‘PRISM\_tmin\_30yr\_normal\_800mM2\_annual\_asc’, ‘PRISM\_vpdmax\_30yr\_normal\_800mM2\_annual\_asc’, and ‘PRISM\_vpdmin\_30yr\_normal\_800mM2\_annual\_asc’.

**Included in Final Model:** Yes (Mean temperature)

**Units:** See documentation: http://www.prism.oregonstate.edu/documents/PRISM\_datasets.pdf

**Original Resolution:** 800 meter x 800 meter

**Dates:** 30 year average

**Source:** PRISM Climate Group: http://www.prism.oregonstate.edu/normals/

**Citation:** PRISM Climate Group. “Oregon State University.” PRISM Gridded Climate Data, 2018. http://www.prism.oregonstate.edu/documents/PRISM\_terms\_of\_use.pdf.

### 10.1.10 PRISM Seasonal Climate Data

**Description:** Using the 30 year average monthly data, we generate seasonal averages for the PRISM climate variables. See section 2.1.3 for more information on how these data were generated.

**Categorical Type:** Climate

**Other Designation:** ‘Pred\_Fall\_ppt’, ‘Pred\_Fall\_tdmean’, ‘Pred\_Fall\_tmax’,‘Pred\_Fall\_tmean’, ‘Pred\_Fall\_tmin’, ‘Pred\_Fall\_vpdmax’, ‘Pred\_Fall\_vpdmin’, ‘Pred\_Spring\_ppt’, ‘Pred\_Spring\_tdmean’, ‘Pred\_Spring\_tmax’, ‘Pred\_Spring\_tmean’, ‘Pred\_Spring\_tmin’, ‘Pred\_Spring\_vpdmax’, ‘Pred\_Spring\_vpdmin’, ‘Pred\_Summer\_ppt’, ‘Pred\_Summer\_tdmean’, ‘Pred\_Summer\_tmax’, ‘Pred\_Summer\_tmean’, ‘Pred\_Summer\_tmin’, ‘Pred\_Summer\_vpdmax’, ‘Pred\_Summer\_vpdmin’, ‘Pred\_Winter\_ppt’, ‘Pred\_Winter\_tdmean’, ‘Pred\_Winter\_tmax’, ‘Pred\_Winter\_tmean’, ‘Pred\_Winter\_tmin’, ‘Pred\_Winter\_vpdmax’, and ‘Pred\_Winter\_vpdmin’.

**Included in Final Model:** No

**Units:** See documentation: http://www.prism.oregonstate.edu/documents/PRISM\_datasets.pdf

**Original Resolution:** 800 meter x 800 meter

**Dates:** 30 year average

**Source:** PRISM Climate Group: http://www.prism.oregonstate.edu/normals/

**Citation:** PRISM Climate Group. “Oregon State University.” PRISM Gridded Climate Data, 2018. http://www.prism.oregonstate.edu/documents/PRISM\_terms\_of\_use.pdf.

### 10.1.11 Cost-distance Data

**Description:** Using the DEM data, we generate the slope raster and then a friction surface. Next, using Tobler’s hiking function, we create a cost-distance surface from lakes, springs, streams, and wetlands.

**Categorical Type:** Resource Distribution

**Other Designation:** ‘lakes\_cd’, ‘springs\_cd’, ‘streams\_cd’, and ‘wetlands\_cd’.

**Included in Final Model:** Yes (Springs, streams, and wetlands)

**Units:** Minutes

**Original Resolution:** 5 meter x 5 meter

**Dates:** NA

**Source:** Utah AGRC

Springs, Streams, and Lakes: https://gis.utah.gov/data/water/lakes-rivers-dams/

Wetlands: https://gis.utah.gov/data/water/wetlands/

**Citation:** Utah AGRC. Utah GIS Portal, 2014.

### 10.1.12 Watershed Size

**Description:** The area of a landscape feature within which water drains to a common outlet.

**Categorical Type:** Landscape

**Other Designation:** ‘wtrshd\_size’

**Included in Final Model:** Yes

**Units:** square meters

**Original Resolution:** Derived from polygon shapefile, but raster is 5 meters x 5 meters.

**Dates:** NA

**Source:** Utah AGRC: https://gis.utah.gov/data/water/watersheds/

**Citation:** Utah AGRC. Utah GIS Portal, 2014.

## 10.2 Site Classification Information

Here, we explain how we classify a site component to the Formative Period.

### 10.2.1 Generic Formative Period Sites

*Def:* Sites consisting of components that show evidence of prehistoric agricultural activities, specifically corn agriculture, including storage features and residential structures, and various ceramic types.

*ID Rule:* If (i) cultural affiliation is Fremont, Anasazi, Basketmaker I-III, or Pueblo I-V, then the site is Formative; OR the site includes Eastgate Expanding Stem, Rose Spring Side-Notched, or Rose Spring Corner -Notched projectile points. Note: Defer to rules for classifying as Fremont, Pueblo, or Basketmaker II where possible.

### 10.2.2 Fremont Sites

*Def:* Sites consisting of components that show evidence of prehistoric agricultural activities, specifically maize agriculture, including storage features (granaries, cists), pit houses, and plain ceramic artifacts or sherds (specifically Emery Gray Ware).

*ID Rule:* If (i) cultural affiliation is Fremont; OR (ii) the site includes pithouses (with few if any above ground residential structures) or storage features (granaries, cists); AND (iii) the site includes Emery Gray Ware ceramics, then the site is Fremont.

### 10.2.3 Pueblo Sites

*Def:* Sites consisting of components that show evidence of prehistoric agricultural activities, specifically maize agriculture, including storage features and residential structures, and various decorated ceramic types (except Emery Gray Ware or Plain BrownWare).

*ID Rule:* If (i) cultural affiliation is Basketmaker III, Anasazi, or Pueblo I-V; OR (ii) the site includes above ground room blocks, kivas, cliff dwellings, granaries, or cists; OR (iii) the site contains Parowan Basal-Notched or Bull Creek PPs; OR (iv) the site contains any complex or decorated ceramic type (basically any ceramic type that is not Brown, Utility, or Emery Gray Ware), then the site is Pueblo.

### 10.2.4 Basketmaker II

*Def:* Sites consisting of components that show evidence of prehistoric agricultural activities, specifically maize agriculture, including storage features and residential structures, but lacking ceramic artifacts or sherds.

*ID Rule:* If (i) cultural affiliation is Basketmaker (general), Basketmaker I2 , or Basketmaker II; OR (ii) the site includes pithouses or storage features; AND (iii) the site includes NO ceramics, then the site is Basketmaker II.

## 10.3 Documentation History

The data in this study date from the 1920s to 2018 and were collected using a number of inventory methods. The majority of the data collected from digital site forms come from a standard recording method, IMACS. The GSENM BLM has made a concerted effort to revisit and re-document archaeological sites not yet recorded according to IMACS standards, so while original documentation for some sites may have occurred prior to the standardized format, most of the database is in an IMACS format.

Prior to (ca.) 1950, there was not a standardized data collection method. Fortunately, many of the reports in the 1920s and 1930s were extremely descriptive and have been relocated and re-recorded on IMACS forms. Beginning in 1948, Jesse Jennings came to the University of Utah and immediately implemented a series of “statewide” surveys to catalog the resources across Utah. His efforts resulted in the first attempt at a standardized Utah site form, which was a two-page form that was entirely descriptive and vague on locations. This form was utilized into the early 1970s. It should also be noted that this two-page form was developed and utilized prior to the emergence of modern type names for ceramics and projectile points, and this makes it very difficult (or speculative) to translate old terms into modern ones.

The emergence of computer databases in the 1970s, and the implementation of federal standards for site documentation in the early 1980s resulted in a regional initiative, also led by Jennings and in partnership with BLM and the United States Forest Service (USFS), to establish uniform documentation standards for the Intermountain West, and to establish a searchable computer database. Nevada and Utah were entirely within the IMACS reporting requirements, and much of Wyoming and Idaho were part of it, as well as a small portion of California. The Intermountain Antiquities Computer System (IMACS) was first rolled out in 1982 and contained five different forms, all with multiple numerical and letter codes for various fields related to environmental, administrative, feature types, artifact types, etc. It also used UTM coordinates whereby sites could be more accurately plotted. The form was mandatory in Utah. In 1992, IMACS was revised and streamlined, but it remained intact until it was replaced in 2020 with a new site form that ironically is not searchable except by optical character recognition.
